# Azimuthal correlations in Z +jets events in proton–proton collisions at $\sqrt{s} = 13\,\text {Te}\hspace{-.08em}\text {V}$

**DOI:** 10.1140/epjc/s10052-023-11833-z

**Published:** 2023-08-11

**Authors:** A. Tumasyan, W. Adam, J. W. Andrejkovic, T. Bergauer, S. Chatterjee, K. Damanakis, M. Dragicevic, A. Escalante Del Valle, R. Frühwirth, M. Jeitler, N. Krammer, L. Lechner, D. Liko, I. Mikulec, P. Paulitsch, F. M. Pitters, J. Schieck, R. Schöfbeck, D. Schwarz, S. Templ, W. Waltenberger, C.-E. Wulz, M. R. Darwish, E. A. De Wolf, T. Janssen, T. Kello, A. Lelek, H. Rejeb Sfar, P. Van Mechelen, S. Van Putte, N. Van Remortel, E. S. Bols, J. D’Hondt, A. De Moor, M. Delcourt, H. El Faham, S. Lowette, S. Moortgat, A. Morton, D. Müller, A. R. Sahasransu, S. Tavernier, W. Van Doninck, D. Vannerom, D. Beghin, B. Bilin, B. Clerbaux, G. De Lentdecker, L. Favart, A. K. Kalsi, K. Lee, M. Mahdavikhorrami, I. Makarenko, S. Paredes, L. Pétré, A. Popov, N. Postiau, E. Starling, L. Thomas, M. Vanden Bemden, C. Vander Velde, P. Vanlaer, T. Cornelis, D. Dobur, J. Knolle, L. Lambrecht, G. Mestdach, M. Niedziela, C. Rendón, C. Roskas, A. Samalan, K. Skovpen, M. Tytgat, N. Van Den Bossche, B. Vermassen, L. Wezenbeek, A. Benecke, A. Bethani, G. Bruno, F. Bury, C. Caputo, P. David, C. Delaere, I. S. Donertas, A. Giammanco, K. Jaffel, Sa. Jain, V. Lemaitre, K. Mondal, J. Prisciandaro, A. Taliercio, M. Teklishyn, T. T. Tran, P. Vischia, S. Wertz, G. A. Alves, C. Hensel, A. Moraes, P. Rebello Teles, W. L. Aldá Júnior, M. Alves Gallo Pereira, M. Barroso Ferreira Filho, H. Brandao Malbouisson, W. Carvalho, J. Chinellato, E. M. Da Costa, G. G. Da Silveira, D. De Jesus Damiao, V. Dos Santos Sousa, S. Fonseca De Souza, J. Martins, C. Mora Herrera, K. Mota Amarilo, L. Mundim, H. Nogima, A. Santoro, S. M. Silva Do Amaral, A. Sznajder, M. Thiel, F. Torres Da Silva De Araujo, A. Vilela Pereira, C. A. Bernardes, L. Calligaris, T. R. Fernandez Perez Tomei, E. M. Gregores, D. S. Lemos, P. G. Mercadante, S. F. Novaes, Sandra S. Padula, A. Aleksandrov, G. Antchev, R. Hadjiiska, P. Iaydjiev, M. Misheva, M. Rodozov, M. Shopova, G. Sultanov, A. Dimitrov, T. Ivanov, L. Litov, B. Pavlov, P. Petkov, A. Petrov, T. Cheng, T. Javaid, M. Mittal, L. Yuan, M. Ahmad, G. Bauer, C. Dozen, Z. Hu, Y. Wang, K. Yi, E. Chapon, G. M. Chen, H. S. Chen, M. Chen, F. Iemmi, A. Kapoor, D. Leggat, H. Liao, Z.-A. Liu, V. Milosevic, F. Monti, R. Sharma, J. Tao, J. Thomas-Wilsker, J. Wang, H. Zhang, J. Zhao, A. Agapitos, Y. An, Y. Ban, C. Chen, A. Levin, Q. Li, X. Lyu, Y. Mao, S. J. Qian, D. Wang, J. Xiao, H. Yang, M. Lu, Z. You, X. Gao, H. Okawa, Y. Zhang, Z. Lin, M. Xiao, C. Avila, A. Cabrera, C. Florez, J. Fraga, J. Mejia Guisao, F. Ramirez, J. D. Ruiz Alvarez, D. Giljanovic, N. Godinovic, D. Lelas, I. Puljak, Z. Antunovic, M. Kovac, T. Sculac, V. Brigljevic, D. Ferencek, D. Majumder, M. Roguljic, A. Starodumov, T. Susa, A. Attikis, K. Christoforou, G. Kole, M. Kolosova, S. Konstantinou, J. Mousa, C. Nicolaou, F. Ptochos, P. A. Razis, H. Rykaczewski, H. Saka, M. Finger, M. Finger, A. Kveton, E. Ayala, E. Carrera Jarrin, S. Abu Zeid, S. Elgammal, M. A. Mahmoud, Y. Mohammed, S. Bhowmik, R. K. Dewanjee, K. Ehataht, M. Kadastik, S. Nandan, C. Nielsen, J. Pata, M. Raidal, L. Tani, C. Veelken, P. Eerola, H. Kirschenmann, K. Osterberg, M. Voutilainen, S. Bharthuar, E. Brücken, F. Garcia, J. Havukainen, M. S. Kim, R. Kinnunen, T. Lampén, K. Lassila-Perini, S. Lehti, T. Lindén, M. Lotti, L. Martikainen, M. Myllymäki, J. Ott, M. m. Rantanen, H. Siikonen, E. Tuominen, J. Tuominiemi, P. Luukka, H. Petrow, T. Tuuva, C. Amendola, M. Besancon, F. Couderc, M. Dejardin, D. Denegri, J. L. Faure, F. Ferri, S. Ganjour, P. Gras, G. Hamel de Monchenault, P. Jarry, B. Lenzi, J. Malcles, J. Rander, A. Rosowsky, M. Ö. Sahin, A. Savoy-Navarro, P. Simkina, M. Titov, G. B. Yu, S. Ahuja, F. Beaudette, M. Bonanomi, A. Buchot Perraguin, P. Busson, A. Cappati, C. Charlot, O. Davignon, B. Diab, G. Falmagne, B. A. Fontana Santos Alves, S. Ghosh, R. Granier de Cassagnac, A. Hakimi, I. Kucher, J. Motta, M. Nguyen, C. Ochando, P. Paganini, J. Rembser, R. Salerno, U. Sarkar, J. B. Sauvan, Y. Sirois, A. Tarabini, A. Zabi, A. Zghiche, J.-L. Agram, J. Andrea, D. Apparu, D. Bloch, G. Bourgatte, J.-M. Brom, E. C. Chabert, C. Collard, D. Darej, J.-C. Fontaine, U. Goerlach, C. Grimault, A.-C. Le Bihan, E. Nibigira, P. Van Hove, E. Asilar, S. Beauceron, C. Bernet, G. Boudoul, C. Camen, A. Carle, N. Chanon, D. Contardo, P. Depasse, H. El Mamouni, J. Fay, S. Gascon, M. Gouzevitch, B. Ille, I. B. Laktineh, H. Lattaud, A. Lesauvage, M. Lethuillier, L. Mirabito, S. Perries, K. Shchablo, V. Sordini, G. Touquet, M. Vander Donckt, S. Viret, I. Bagaturia, I. Lomidze, Z. Tsamalaidze, V. Botta, L. Feld, K. Klein, M. Lipinski, D. Meuser, A. Pauls, N. Röwert, J. Schulz, M. Teroerde, A. Dodonova, N. Eich, D. Eliseev, M. Erdmann, P. Fackeldey, B. Fischer, T. Hebbeker, K. Hoepfner, F. Ivone, L. Mastrolorenzo, M. Merschmeyer, A. Meyer, G. Mocellin, S. Mondal, S. Mukherjee, D. Noll, A. Novak, A. Pozdnyakov, Y. Rath, H. Reithler, A. Schmidt, S. C. Schuler, A. Sharma, L. Vigilante, S. Wiedenbeck, S. Zaleski, C. Dziwok, G. Flügge, W. Haj Ahmad, O. Hlushchenko, T. Kress, A. Nowack, O. Pooth, D. Roy, A. Stahl, T. Ziemons, A. Zotz, H. Aarup Petersen, M. Aldaya Martin, P. Asmuss, S. Baxter, M. Bayatmakou, O. Behnke, A. Bermúdez Martínez, S. Bhattacharya, A. A. Bin Anuar, F. Blekman, K. Borras, D. Brunner, A. Campbell, A. Cardini, C. Cheng, F. Colombina, S. Consuegra Rodríguez, G. Correia Silva, M. De Silva, L. Didukh, G. Eckerlin, D. Eckstein, L. I. Estevez Banos, O. Filatov, E. Gallo, A. Geiser, A. Giraldi, G. Greau, A. Grohsjean, M. Guthoff, A. Jafari, N. Z. Jomhari, A. Kasem, M. Kasemann, H. Kaveh, C. Kleinwort, R. Kogler, D. Krücker, W. Lange, K. Lipka, W. Lohmann, R. Mankel, I.-A. Melzer-Pellmann, M. Mendizabal Morentin, J. Metwally, A. B. Meyer, M. Meyer, J. Mnich, A. Mussgiller, A. Nürnberg, Y. Otarid, D. Pérez Adán, D. Pitzl, A. Raspereza, B. Ribeiro Lopes, J. Rübenach, A. Saggio, A. Saibel, M. Savitskyi, M. Scham, V. Scheurer, S. Schnake, P. Schütze, C. Schwanenberger, M. Shchedrolosiev, R. E. Sosa Ricardo, D. Stafford, N. Tonon, M. Van De Klundert, F. Vazzoler, R. Walsh, D. Walter, Q. Wang, Y. Wen, K. Wichmann, L. Wiens, C. Wissing, S. Wuchterl, R. Aggleton, S. Albrecht, S. Bein, L. Benato, P. Connor, K. De Leo, M. Eich, K. El Morabit, F. Feindt, A. Fröhlich, C. Garbers, E. Garutti, P. Gunnellini, M. Hajheidari, J. Haller, A. Hinzmann, G. Kasieczka, R. Klanner, T. Kramer, V. Kutzner, J. Lange, T. Lange, A. Lobanov, A. Malara, C. Matthies, A. Mehta, L. Moureaux, A. Nigamova, K. J. Pena Rodriguez, M. Rieger, O. Rieger, P. Schleper, M. Schröder, J. Schwandt, J. Sonneveld, H. Stadie, G. Steinbrück, A. Tews, I. Zoi, J. Bechtel, S. Brommer, M. Burkart, E. Butz, R. Caspart, T. Chwalek, W. De Boer, A. Dierlamm, A. Droll, N. Faltermann, M. Giffels, J. O. Gosewisch, A. Gottmann, F. Hartmann, C. Heidecker, U. Husemann, P. Keicher, R. Koppenhöfer, S. Maier, S. Mitra, Th. Müller, M. Neukum, G. Quast, K. Rabbertz, J. Rauser, D. Savoiu, M. Schnepf, D. Seith, I. Shvetsov, H. J. Simonis, R. Ulrich, J. Van Der Linden, R. F. Von Cube, M. Wassmer, M. Weber, S. Wieland, R. Wolf, S. Wozniewski, S. Wunsch, G. Anagnostou, G. Daskalakis, A. Kyriakis, A. Stakia, M. Diamantopoulou, D. Karasavvas, P. Kontaxakis, C. K. Koraka, A. Manousakis-Katsikakis, A. Panagiotou, I. Papavergou, N. Saoulidou, K. Theofilatos, E. Tziaferi, K. Vellidis, E. Vourliotis, G. Bakas, K. Kousouris, I. Papakrivopoulos, G. Tsipolitis, A. Zacharopoulou, K. Adamidis, I. Bestintzanos, I. Evangelou, C. Foudas, P. Gianneios, P. Katsoulis, P. Kokkas, N. Manthos, I. Papadopoulos, J. Strologas, M. Csanád, K. Farkas, M. M. A. Gadallah, S. Lökös, P. Major, K. Mandal, G. Pásztor, A. J. Rádl, O. Surányi, G. I. Veres, M. Bartók, G. Bencze, C. Hajdu, D. Horvath, F. Sikler, V. Veszpremi, S. Czellar, D. Fasanella, F. Fienga, J. Karancsi, J. Molnar, Z. Szillasi, D. Teyssier, P. Raics, Z. L. Trocsanyi, B. Ujvari, T. Csorgo, F. Nemes, T. Novak, S. Bansal, S. B. Beri, V. Bhatnagar, G. Chaudhary, S. Chauhan, N. Dhingra, R. Gupta, A. Kaur, H. Kaur, M. Kaur, P. Kumari, M. Meena, K. Sandeep, J. B. Singh, A. K. Virdi, A. Ahmed, A. Bhardwaj, B. C. Choudhary, M. Gola, S. Keshri, A. Kumar, M. Naimuddin, P. Priyanka, K. Ranjan, S. Saumya, A. Shah, M. Bharti, R. Bhattacharya, S. Bhattacharya, D. Bhowmik, S. Dutta, S. Dutta, B. Gomber, M. Maity, P. Palit, P. K. Rout, G. Saha, B. Sahu, S. Sarkar, M. Sharan, P. K. Behera, S. C. Behera, P. Kalbhor, J. R. Komaragiri, D. Kumar, A. Muhammad, L. Panwar, R. Pradhan, P. R. Pujahari, A. Sharma, A. K. Sikdar, P. C. Tiwari, K. Naskar, T. Aziz, S. Dugad, M. Kumar, G. B. Mohanty, S. Banerjee, R. Chudasama, M. Guchait, S. Karmakar, S. Kumar, G. Majumder, K. Mazumdar, S. Mukherjee, S. Bahinipati, C. Kar, P. Mal, T. Mishra, V. K. Muraleedharan Nair Bindhu, A. Nayak, P. Saha, N. Sur, S. K. Swain, D. Vats, A. Alpana, S. Dube, B. Kansal, A. Laha, S. Pandey, A. Rastogi, S. Sharma, H. Bakhshiansohi, E. Khazaie, M. Zeinali, S. Chenarani, S. M. Etesami, M. Khakzad, M. Mohammadi Najafabadi, M. Grunewald, M. Abbrescia, R. Aly, C. Aruta, A. Colaleo, D. Creanza, N. De Filippis, M. De Palma, A. Di Florio, A. Di Pilato, W. Elmetenawee, F. Errico, L. Fiore, G. Iaselli, M. Ince, S. Lezki, G. Maggi, M. Maggi, I. Margjeka, V. Mastrapasqua, S. My, S. Nuzzo, A. Pellecchia, A. Pompili, G. Pugliese, D. Ramos, A. Ranieri, G. Selvaggi, L. Silvestris, F. M. Simone, Ü. Sözbilir, R. Venditti, P. Verwilligen, G. Abbiendi, C. Battilana, D. Bonacorsi, L. Borgonovi, L. Brigliadori, R. Campanini, P. Capiluppi, A. Castro, F. R. Cavallo, C. Ciocca, M. Cuffiani, G. M. Dallavalle, T. Diotalevi, F. Fabbri, A. Fanfani, P. Giacomelli, L. Giommi, C. Grandi, L. Guiducci, S. Lo Meo, L. Lunerti, S. Marcellini, G. Masetti, F. L. Navarria, A. Perrotta, F. Primavera, A. M. Rossi, T. Rovelli, G. P. Siroli, S. Albergo, S. Costa, A. Di Mattia, R. Potenza, A. Tricomi, C. Tuve, G. Barbagli, A. Cassese, R. Ceccarelli, V. Ciulli, C. Civinini, R. D’Alessandro, E. Focardi, G. Latino, P. Lenzi, M. Lizzo, M. Meschini, S. Paoletti, R. Seidita, G. Sguazzoni, L. Viliani, L. Benussi, S. Bianco, D. Piccolo, M. Bozzo, F. Ferro, R. Mulargia, E. Robutti, S. Tosi, A. Benaglia, G. Boldrini, F. Brivio, F. Cetorelli, F. De Guio, M. E. Dinardo, P. Dini, S. Gennai, A. Ghezzi, P. Govoni, L. Guzzi, M. T. Lucchini, M. Malberti, S. Malvezzi, A. Massironi, D. Menasce, L. Moroni, M. Paganoni, D. Pedrini, B. S. Pinolini, S. Ragazzi, N. Redaelli, T. Tabarelli de Fatis, D. Valsecchi, D. Zuolo, S. Buontempo, F. Carnevali, N. Cavallo, A. De Iorio, F. Fabozzi, A. O. M. Iorio, L. Lista, S. Meola, P. Paolucci, B. Rossi, C. Sciacca, P. Azzi, N. Bacchetta, D. Bisello, P. Bortignon, A. Bragagnolo, R. Carlin, P. Checchia, T. Dorigo, U. Dosselli, F. Gasparini, U. Gasparini, G. Grosso, L. Layer, E. Lusiani, M. Margoni, F. Marini, A. T. Meneguzzo, J. Pazzini, P. Ronchese, R. Rossin, F. Simonetto, G. Strong, M. Tosi, H. Yarar, M. Zanetti, P. Zotto, A. Zucchetta, G. Zumerle, C. Aimè, A. Braghieri, S. Calzaferri, D. Fiorina, P. Montagna, S. P. Ratti, V. Re, C. Riccardi, P. Salvini, I. Vai, P. Vitulo, P. Asenov, G. M. Bilei, D. Ciangottini, L. Fanò, M. Magherini, G. Mantovani, V. Mariani, M. Menichelli, F. Moscatelli, A. Piccinelli, M. Presilla, A. Rossi, A. Santocchia, D. Spiga, T. Tedeschi, P. Azzurri, G. Bagliesi, V. Bertacchi, L. Bianchini, T. Boccali, E. Bossini, R. Castaldi, M. A. Ciocci, V. D’Amante, R. Dell’Orso, M. R. Di Domenico, S. Donato, A. Giassi, F. Ligabue, E. Manca, G. Mandorli, D. Matos Figueiredo, A. Messineo, M. Musich, F. Palla, S. Parolia, G. Ramirez-Sanchez, A. Rizzi, G. Rolandi, S. Roy Chowdhury, A. Scribano, N. Shafiei, P. Spagnolo, R. Tenchini, G. Tonelli, N. Turini, A. Venturi, P. G. Verdini, P. Barria, M. Campana, F. Cavallari, D. Del Re, E. Di Marco, M. Diemoz, E. Longo, P. Meridiani, G. Organtini, F. Pandolfi, R. Paramatti, C. Quaranta, S. Rahatlou, C. Rovelli, F. Santanastasio, L. Soffi, R. Tramontano, N. Amapane, R. Arcidiacono, S. Argiro, M. Arneodo, N. Bartosik, R. Bellan, A. Bellora, J. Berenguer Antequera, C. Biino, N. Cartiglia, M. Costa, R. Covarelli, N. Demaria, M. Grippo, B. Kiani, F. Legger, C. Mariotti, S. Maselli, A. Mecca, E. Migliore, E. Monteil, M. Monteno, M. M. Obertino, G. Ortona, L. Pacher, N. Pastrone, M. Pelliccioni, M. Ruspa, K. Shchelina, F. Siviero, V. Sola, A. Solano, D. Soldi, A. Staiano, M. Tornago, D. Trocino, G. Umoret, A. Vagnerini, S. Belforte, V. Candelise, M. Casarsa, F. Cossutti, A. Da Rold, G. Della Ricca, G. Sorrentino, S. Dogra, C. Huh, B. Kim, D. H. Kim, G. N. Kim, J. Kim, J. Lee, S. W. Lee, C. S. Moon, Y. D. Oh, S. I. Pak, S. Sekmen, Y. C. Yang, H. Kim, D. H. Moon, B. Francois, T. J. Kim, J. Park, S. Cho, S. Choi, B. Hong, K. Lee, K. S. Lee, J. Lim, J. Park, S. K. Park, J. Yoo, J. Goh, A. Gurtu, H. S. Kim, Y. Kim, J. Almond, J. H. Bhyun, J. Choi, S. Jeon, J. Kim, J. S. Kim, S. Ko, H. Kwon, H. Lee, S. Lee, B. H. Oh, M. Oh, S. B. Oh, H. Seo, U. K. Yang, I. Yoon, W. Jang, D. Y. Kang, Y. Kang, S. Kim, B. Ko, J. S. H. Lee, Y. Lee, J. A. Merlin, I. C. Park, Y. Roh, M. S. Ryu, D. Song, I. J. Watson, S. Yang, S. Ha, H. D. Yoo, M. Choi, H. Lee, Y. Lee, I. Yu, T. Beyrouthy, Y. Maghrbi, K. Dreimanis, V. Veckalns, M. Ambrozas, A. Carvalho Antunes De Oliveira, A. Juodagalvis, A. Rinkevicius, G. Tamulaitis, N. Bin Norjoharuddeen, S. Y. Hoh, Z. Zolkapli, J. F. Benitez, A. Castaneda Hernandez, H. A. Encinas Acosta, L. G. Gallegos Maríñez, M. León Coello, J. A. Murillo Quijada, A. Sehrawat, L. Valencia Palomo, G. Ayala, H. Castilla-Valdez, E. De La Cruz-Burelo, I. Heredia-De La Cruz, R. Lopez-Fernandez, C. A. Mondragon Herrera, D. A. Perez Navarro, R. Reyes-Almanza, A. Sánchez Hernández, S. Carrillo Moreno, C. Oropeza Barrera, F. Vazquez Valencia, I. Pedraza, H. A. Salazar Ibarguen, C. Uribe Estrada, I. Bubanja, J. Mijuskovic, N. Raicevic, D. Krofcheck, P. H. Butler, A. Ahmad, M. I. Asghar, A. Awais, M. I. M. Awan, M. Gul, H. R. Hoorani, W. A. Khan, M. A. Shah, M. Shoaib, M. Waqas, V. Avati, L. Grzanka, M. Malawski, H. Bialkowska, M. Bluj, B. Boimska, M. Górski, M. Kazana, M. Szleper, P. Zalewski, K. Bunkowski, K. Doroba, A. Kalinowski, M. Konecki, J. Krolikowski, M. Araujo, P. Bargassa, D. Bastos, A. Boletti, P. Faccioli, M. Gallinaro, J. Hollar, N. Leonardo, T. Niknejad, M. Pisano, J. Seixas, O. Toldaiev, J. Varela, P. Adzic, M. Dordevic, P. Milenovic, J. Milosevic, M. Aguilar-Benitez, J. Alcaraz Maestre, A. Álvarez Fernández, I. Bachiller, M. Barrio Luna, Cristina F. Bedoya, C. A. Carrillo Montoya, M. Cepeda, M. Cerrada, N. Colino, B. De La Cruz, A. Delgado Peris, J. P. Fernández Ramos, J. Flix, M. C. Fouz, O. Gonzalez Lopez, S. Goy Lopez, J. M. Hernandez, M. I. Josa, J. León Holgado, D. Moran, Á. Navarro Tobar, C. Perez Dengra, A. Pérez-Calero Yzquierdo, J. Puerta Pelayo, I. Redondo, L. Romero, S. Sánchez Navas, L. Urda Gómez, C. Willmott, J. F. de Trocóniz, B. Alvarez Gonzalez, J. Cuevas, J. Fernandez Menendez, S. Folgueras, I. Gonzalez Caballero, J. R. González Fernández, E. Palencia Cortezon, C. Ramón Álvarez, V. Rodríguez Bouza, A. Soto Rodríguez, A. Trapote, N. Trevisani, C. Vico Villalba, J. A. Brochero Cifuentes, I. J. Cabrillo, A. Calderon, J. Duarte Campderros, M. Fernandez, C. Fernandez Madrazo, P. J. Fernández Manteca, A. García Alonso, G. Gomez, C. Martinez Rivero, P. Martinez Ruiz del Arbol, F. Matorras, P. Matorras Cuevas, J. Piedra Gomez, C. Prieels, A. Ruiz-Jimeno, L. Scodellaro, I. Vila, J. M. Vizan Garcia, M. K. Jayananda, B. Kailasapathy, D. U. J. Sonnadara, D. D. C. Wickramarathna, W. G. D. Dharmaratna, K. Liyanage, N. Perera, N. Wickramage, T. K. Aarrestad, D. Abbaneo, J. Alimena, E. Auffray, G. Auzinger, J. Baechler, P. Baillon, D. Barney, J. Bendavid, M. Bianco, A. Bocci, C. Caillol, T. Camporesi, M. Capeans Garrido, G. Cerminara, N. Chernyavskaya, S. S. Chhibra, S. Choudhury, M. Cipriani, L. Cristella, D. d’Enterria, A. Dabrowski, A. David, A. De Roeck, M. M. Defranchis, M. Deile, M. Dobson, M. Dünser, N. Dupont, A. Elliott-Peisert, F. Fallavollita, A. Florent, L. Forthomme, G. Franzoni, W. Funk, S. Ghosh, S. Giani, D. Gigi, K. Gill, F. Glege, L. Gouskos, E. Govorkova, M. Haranko, J. Hegeman, V. Innocente, T. James, P. Janot, J. Kaspar, J. Kieseler, M. Komm, N. Kratochwil, C. Lange, S. Laurila, P. Lecoq, A. Lintuluoto, C. Lourenço, B. Maier, L. Malgeri, S. Mallios, M. Mannelli, A. C. Marini, F. Meijers, S. Mersi, E. Meschi, F. Moortgat, M. Mulders, S. Orfanelli, L. Orsini, F. Pantaleo, E. Perez, M. Peruzzi, A. Petrilli, G. Petrucciani, A. Pfeiffer, M. Pierini, D. Piparo, M. Pitt, H. Qu, T. Quast, D. Rabady, A. Racz, G. Reales Gutiérrez, M. Rovere, H. Sakulin, J. Salfeld-Nebgen, S. Scarfi, M. Selvaggi, A. Sharma, P. Silva, W. Snoeys, P. Sphicas, S. Summers, K. Tatar, V. R. Tavolaro, D. Treille, P. Tropea, A. Tsirou, J. Wanczyk, K. A. Wozniak, W. D. Zeuner, L. Caminada, A. Ebrahimi, W. Erdmann, R. Horisberger, Q. Ingram, H. C. Kaestli, D. Kotlinski, M. Missiroli, L. Noehte, T. Rohe, K. Androsov, M. Backhaus, P. Berger, A. Calandri, A. De Cosa, G. Dissertori, M. Dittmar, M. Donegà, C. Dorfer, F. Eble, K. Gedia, F. Glessgen, T. A. Gómez Espinosa, C. Grab, D. Hits, W. Lustermann, A.-M. Lyon, R. A. Manzoni, L. Marchese, C. Martin Perez, M. T. Meinhard, F. Nessi-Tedaldi, J. Niedziela, F. Pauss, V. Perovic, S. Pigazzini, M. G. Ratti, M. Reichmann, C. Reissel, T. Reitenspiess, B. Ristic, D. Ruini, D. A. Sanz Becerra, V. Stampf, J. Steggemann, R. Wallny, C. Amsler, P. Bärtschi, C. Botta, D. Brzhechko, M. F. Canelli, K. Cormier, A. De Wit, R. Del Burgo, J. K. Heikkilä, M. Huwiler, W. Jin, A. Jofrehei, B. Kilminster, S. Leontsinis, S. P. Liechti, A. Macchiolo, P. Meiring, V. M. Mikuni, U. Molinatti, I. Neutelings, A. Reimers, P. Robmann, S. Sanchez Cruz, K. Schweiger, M. Senger, Y. Takahashi, C. Adloff, C. M. Kuo, W. Lin, A. Roy, T. Sarkar, S. S. Yu, L. Ceard, Y. Chao, K. F. Chen, P. H. Chen, P. s. Chen, H. Cheng, W.-S. Hou, Y. y. Li, R.-S. Lu, E. Paganis, A. Psallidas, A. Steen, H. y. Wu, E. Yazgan, P. r. Yu, B. Asavapibhop, C. Asawatangtrakuldee, N. Srimanobhas, F. Boran, S. Damarseckin, Z. S. Demiroglu, F. Dolek, I. Dumanoglu, E. Eskut, Y. Guler, E. Gurpinar Guler, C. Isik, O. Kara, A. Kayis Topaksu, U. Kiminsu, G. Onengut, K. Ozdemir, A. Polatoz, A. E. Simsek, B. Tali, U. G. Tok, S. Turkcapar, I. S. Zorbakir, G. Karapinar, K. Ocalan, M. Yalvac, B. Akgun, I. O. Atakisi, E. Gülmez, M. Kaya, O. Kaya, Ö. Özçelik, S. Tekten, E. A. Yetkin, A. Cakir, K. Cankocak, Y. Komurcu, S. Sen, S. Cerci, I. Hos, B. Isildak, B. Kaynak, S. Ozkorucuklu, H. Sert, C. Simsek, D. Sunar Cerci, C. Zorbilmez, B. Grynyov, L. Levchuk, D. Anthony, E. Bhal, S. Bologna, J. J. Brooke, A. Bundock, E. Clement, D. Cussans, H. Flacher, M. Glowacki, J. Goldstein, G. P. Heath, H. F. Heath, L. Kreczko, B. Krikler, S. Paramesvaran, S. Seif El Nasr-Storey, V. J. Smith, N. Stylianou, K. Walkingshaw Pass, R. White, K. W. Bell, A. Belyaev, C. Brew, R. M. Brown, D. J. A. Cockerill, C. Cooke, K. V. Ellis, K. Harder, S. Harper, M.-L. Holmberg, J. Linacre, K. Manolopoulos, D. M. Newbold, E. Olaiya, D. Petyt, T. Reis, T. Schuh, C. H. Shepherd-Themistocleous, I. R. Tomalin, T. Williams, R. Bainbridge, P. Bloch, S. Bonomally, J. Borg, S. Breeze, O. Buchmuller, V. Cepaitis, G. S. Chahal, D. Colling, P. Dauncey, G. Davies, M. Della Negra, S. Fayer, G. Fedi, G. Hall, M. H. Hassanshahi, G. Iles, J. Langford, L. Lyons, A.-M. Magnan, S. Malik, A. Martelli, D. G. Monk, J. Nash, M. Pesaresi, B. C. Radburn-Smith, D. M. Raymond, A. Richards, A. Rose, E. Scott, C. Seez, A. Shtipliyski, A. Tapper, K. Uchida, T. Virdee, M. Vojinovic, N. Wardle, S. N. Webb, D. Winterbottom, K. Coldham, J. E. Cole, A. Khan, P. Kyberd, I. D. Reid, L. Teodorescu, S. Zahid, S. Abdullin, A. Brinkerhoff, B. Caraway, J. Dittmann, K. Hatakeyama, A. R. Kanuganti, B. McMaster, M. Saunders, S. Sawant, C. Sutantawibul, J. Wilson, R. Bartek, A. Dominguez, R. Uniyal, A. M. Vargas Hernandez, A. Buccilli, S. I. Cooper, D. Di Croce, S. V. Gleyzer, C. Henderson, C. U. Perez, P. Rumerio, C. West, A. Akpinar, A. Albert, D. Arcaro, C. Cosby, Z. Demiragli, C. Erice, E. Fontanesi, D. Gastler, S. May, J. Rohlf, K. Salyer, D. Sperka, D. Spitzbart, I. Suarez, A. Tsatsos, S. Yuan, D. Zou, G. Benelli, B. Burkle, X. Coubez, D. Cutts, M. Hadley, U. Heintz, J. M. Hogan, T. Kwon, G. Landsberg, K. T. Lau, D. Li, M. Lukasik, J. Luo, M. Narain, N. Pervan, S. Sagir, F. Simpson, E. Usai, W. Y. Wong, X. Yan, D. Yu, W. Zhang, J. Bonilla, C. Brainerd, R. Breedon, M. Calderon De La Barca Sanchez, M. Chertok, J. Conway, P. T. Cox, R. Erbacher, G. Haza, F. Jensen, O. Kukral, R. Lander, M. Mulhearn, D. Pellett, B. Regnery, D. Taylor, Y. Yao, F. Zhang, M. Bachtis, R. Cousins, A. Datta, D. Hamilton, J. Hauser, M. Ignatenko, M. A. Iqbal, T. Lam, W. A. Nash, S. Regnard, D. Saltzberg, B. Stone, V. Valuev, Y. Chen, R. Clare, J. W. Gary, M. Gordon, G. Hanson, G. Karapostoli, O. R. Long, N. Manganelli, W. Si, S. Wimpenny, Y. Zhang, J. G. Branson, P. Chang, S. Cittolin, S. Cooperstein, D. Diaz, J. Duarte, R. Gerosa, L. Giannini, J. Guiang, R. Kansal, V. Krutelyov, R. Lee, J. Letts, M. Masciovecchio, F. Mokhtar, M. Pieri, B. V. Sathia Narayanan, V. Sharma, M. Tadel, F. Würthwein, Y. Xiang, A. Yagil, N. Amin, C. Campagnari, M. Citron, G. Collura, A. Dorsett, V. Dutta, J. Incandela, M. Kilpatrick, J. Kim, B. Marsh, H. Mei, M. Oshiro, M. Quinnan, J. Richman, U. Sarica, F. Setti, J. Sheplock, P. Siddireddy, D. Stuart, S. Wang, A. Bornheim, O. Cerri, I. Dutta, J. M. Lawhorn, N. Lu, J. Mao, H. B. Newman, T. Q. Nguyen, M. Spiropulu, J. R. Vlimant, C. Wang, S. Xie, Z. Zhang, R. Y. Zhu, J. Alison, S. An, M. B. Andrews, P. Bryant, T. Ferguson, A. Harilal, C. Liu, T. Mudholkar, M. Paulini, A. Sanchez, W. Terrill, J. P. Cumalat, W. T. Ford, A. Hassani, G. Karathanasis, E. MacDonald, R. Patel, A. Perloff, C. Savard, N. Schonbeck, K. Stenson, K. A. Ulmer, S. R. Wagner, N. Zipper, J. Alexander, S. Bright-Thonney, X. Chen, Y. Cheng, D. J. Cranshaw, X. Fan, S. Hogan, J. Monroy, J. R. Patterson, D. Quach, J. Reichert, M. Reid, A. Ryd, W. Sun, J. Thom, P. Wittich, R. Zou, M. Albrow, M. Alyari, G. Apollinari, A. Apresyan, A. Apyan, L. A. T. Bauerdick, D. Berry, J. Berryhill, P. C. Bhat, K. Burkett, J. N. Butler, A. Canepa, G. B. Cerati, H. W. K. Cheung, F. Chlebana, K. F. Di Petrillo, J. Dickinson, V. D. Elvira, Y. Feng, J. Freeman, A. Gandrakota, Z. Gecse, L. Gray, D. Green, S. Grünendahl, O. Gutsche, R. M. Harris, R. Heller, T. C. Herwig, J. Hirschauer, B. Jayatilaka, S. Jindariani, M. Johnson, U. Joshi, T. Klijnsma, B. Klima, K. H. M. Kwok, S. Lammel, D. Lincoln, R. Lipton, T. Liu, C. Madrid, K. Maeshima, C. Mantilla, D. Mason, P. McBride, P. Merkel, S. Mrenna, S. Nahn, J. Ngadiuba, V. Papadimitriou, N. Pastika, K. Pedro, C. Pena, F. Ravera, A. Reinsvold Hall, L. Ristori, E. Sexton-Kennedy, N. Smith, A. Soha, L. Spiegel, J. Strait, L. Taylor, S. Tkaczyk, N. V. Tran, L. Uplegger, E. W. Vaandering, H. A. Weber, P. Avery, D. Bourilkov, L. Cadamuro, V. Cherepanov, R. D. Field, D. Guerrero, M. Kim, E. Koenig, J. Konigsberg, A. Korytov, K. H. Lo, K. Matchev, N. Menendez, G. Mitselmakher, A. Muthirakalayil Madhu, N. Rawal, D. Rosenzweig, S. Rosenzweig, K. Shi, J. Wang, Z. Wu, E. Yigitbasi, X. Zuo, T. Adams, A. Askew, R. Habibullah, V. Hagopian, K. F. Johnson, R. Khurana, T. Kolberg, G. Martinez, H. Prosper, C. Schiber, O. Viazlo, R. Yohay, J. Zhang, M. M. Baarmand, S. Butalla, T. Elkafrawy, M. Hohlmann, R. Kumar Verma, D. Noonan, M. Rahmani, F. Yumiceva, M. R. Adams, H. Becerril Gonzalez, R. Cavanaugh, S. Dittmer, O. Evdokimov, C. E. Gerber, D. J. Hofman, A. H. Merrit, C. Mills, G. Oh, T. Roy, S. Rudrabhatla, M. B. Tonjes, N. Varelas, J. Viinikainen, X. Wang, Z. Ye, M. Alhusseini, K. Dilsiz, L. Emediato, R. P. Gandrajula, O. K. Köseyan, J.-P. Merlo, A. Mestvirishvili, J. Nachtman, H. Ogul, Y. Onel, A. Penzo, C. Snyder, E. Tiras, O. Amram, B. Blumenfeld, L. Corcodilos, J. Davis, A. V. Gritsan, S. Kyriacou, P. Maksimovic, J. Roskes, M. Swartz, T. Á. Vámi, A. Abreu, J. Anguiano, C. Baldenegro Barrera, P. Baringer, A. Bean, Z. Flowers, T. Isidori, S. Khalil, J. King, G. Krintiras, A. Kropivnitskaya, M. Lazarovits, C. Le Mahieu, C. Lindsey, J. Marquez, N. Minafra, M. Murray, M. Nickel, C. Rogan, C. Royon, R. Salvatico, S. Sanders, E. Schmitz, C. Smith, Q. Wang, Z. Warner, J. Williams, G. Wilson, S. Duric, A. Ivanov, K. Kaadze, D. Kim, Y. Maravin, T. Mitchell, A. Modak, K. Nam, F. Rebassoo, D. Wright, E. Adams, A. Baden, O. Baron, A. Belloni, S. C. Eno, N. J. Hadley, S. Jabeen, R. G. Kellogg, T. Koeth, Y. Lai, S. Lascio, A. C. Mignerey, S. Nabili, C. Palmer, M. Seidel, A. Skuja, L. Wang, K. Wong, D. Abercrombie, G. Andreassi, R. Bi, W. Busza, I. A. Cali, Y. Chen, M. D’Alfonso, J. Eysermans, C. Freer, G. Gomez-Ceballos, M. Goncharov, P. Harris, M. Hu, M. Klute, D. Kovalskyi, J. Krupa, Y.-J. Lee, K. Long, C. Mironov, C. Paus, D. Rankin, C. Roland, G. Roland, Z. Shi, G. S. F. Stephans, J. Wang, Z. Wang, B. Wyslouch, R. M. Chatterjee, A. Evans, J. Hiltbrand, Sh. Jain, B. M. Joshi, M. Krohn, Y. Kubota, J. Mans, M. Revering, R. Rusack, R. Saradhy, N. Schroeder, N. Strobbe, M. A. Wadud, K. Bloom, M. Bryson, S. Chauhan, D. R. Claes, C. Fangmeier, L. Finco, F. Golf, C. Joo, I. Kravchenko, I. Reed, J. E. Siado, G. R. Snow, W. Tabb, A. Wightman, F. Yan, A. G. Zecchinelli, G. Agarwal, H. Bandyopadhyay, L. Hay, I. Iashvili, A. Kharchilava, C. McLean, D. Nguyen, J. Pekkanen, S. Rappoccio, A. Williams, G. Alverson, E. Barberis, Y. Haddad, Y. Han, A. Hortiangtham, A. Krishna, J. Li, J. Lidrych, G. Madigan, B. Marzocchi, D. M. Morse, V. Nguyen, T. Orimoto, A. Parker, L. Skinnari, A. Tishelman-Charny, T. Wamorkar, B. Wang, A. Wisecarver, D. Wood, S. Bhattacharya, J. Bueghly, Z. Chen, A. Gilbert, T. Gunter, K. A. Hahn, Y. Liu, N. Odell, M. H. Schmitt, M. Velasco, R. Band, R. Bucci, M. Cremonesi, A. Das, N. Dev, R. Goldouzian, M. Hildreth, K. Hurtado Anampa, C. Jessop, K. Lannon, J. Lawrence, N. Loukas, L. Lutton, J. Mariano, N. Marinelli, I. Mcalister, T. McCauley, C. Mcgrady, K. Mohrman, C. Moore, Y. Musienko, R. Ruchti, A. Townsend, M. Wayne, M. Zarucki, L. Zygala, B. Bylsma, L. S. Durkin, B. Francis, C. Hill, M. Nunez Ornelas, K. Wei, B. L. Winer, B. R. Yates, F. M. Addesa, B. Bonham, P. Das, G. Dezoort, P. Elmer, A. Frankenthal, B. Greenberg, N. Haubrich, S. Higginbotham, A. Kalogeropoulos, G. Kopp, S. Kwan, D. Lange, D. Marlow, K. Mei, I. Ojalvo, J. Olsen, D. Stickland, C. Tully, S. Malik, S. Norberg, A. S. Bakshi, V. E. Barnes, R. Chawla, S. Das, L. Gutay, M. Jones, A. W. Jung, D. Kondratyev, A. M. Koshy, M. Liu, G. Negro, N. Neumeister, G. Paspalaki, S. Piperov, A. Purohit, J. F. Schulte, M. Stojanovic, J. Thieman, F. Wang, R. Xiao, W. Xie, J. Dolen, N. Parashar, D. Acosta, A. Baty, T. Carnahan, M. Decaro, S. Dildick, K. M. Ecklund, S. Freed, P. Gardner, F. J. M. Geurts, A. Kumar, W. Li, B. P. Padley, R. Redjimi, J. Rotter, W. Shi, A. G. Stahl Leiton, S. Yang, L. Zhang, Y. Zhang, A. Bodek, P. de Barbaro, R. Demina, J. L. Dulemba, C. Fallon, T. Ferbel, M. Galanti, A. Garcia-Bellido, O. Hindrichs, A. Khukhunaishvili, E. Ranken, R. Taus, G. P. Van Onsem, K. Goulianos, B. Chiarito, J. P. Chou, Y. Gershtein, E. Halkiadakis, A. Hart, M. Heindl, O. Karacheban, I. Laflotte, A. Lath, R. Montalvo, K. Nash, M. Osherson, S. Salur, S. Schnetzer, S. Somalwar, R. Stone, S. A. Thayil, S. Thomas, H. Wang, H. Acharya, A. G. Delannoy, S. Fiorendi, T. Holmes, S. Spanier, O. Bouhali, M. Dalchenko, A. Delgado, R. Eusebi, J. Gilmore, T. Huang, T. Kamon, H. Kim, S. Luo, S. Malhotra, R. Mueller, D. Overton, D. Rathjens, A. Safonov, N. Akchurin, J. Damgov, V. Hegde, K. Lamichhane, S. W. Lee, T. Mengke, S. Muthumuni, T. Peltola, I. Volobouev, Z. Wang, A. Whitbeck, E. Appelt, S. Greene, A. Gurrola, W. Johns, A. Melo, K. Padeken, F. Romeo, P. Sheldon, S. Tuo, J. Velkovska, M. W. Arenton, B. Cardwell, B. Cox, G. Cummings, J. Hakala, R. Hirosky, M. Joyce, A. Ledovskoy, A. Li, C. Neu, C. E. Perez Lara, B. Tannenwald, S. White, N. Poudyal, S. Banerjee, K. Black, T. Bose, S. Dasu, I. De Bruyn, P. Everaerts, C. Galloni, H. He, M. Herndon, A. Herve, U. Hussain, A. Lanaro, A. Loeliger, R. Loveless, J. Madhusudanan Sreekala, A. Mallampalli, A. Mohammadi, D. Pinna, A. Savin, V. Shang, V. Sharma, W. H. Smith, D. Teague, S. Trembath-Reichert, W. Vetens, S. Afanasiev, V. Andreev, Yu. Andreev, T. Aushev, M. Azarkin, A. Babaev, A. Belyaev, V. Blinov, E. Boos, V. Borshch, D. Budkouski, O. Bychkova, V. Chekhovsky, R. Chistov, M. Danilov, A. Dermenev, T. Dimova, I. Dremin, M. Dubinin, L. Dudko, V. Epshteyn, G. Gavrilov, V. Gavrilov, S. Gninenko, V. Golovtcov, N. Golubev, I. Golutvin, I. Gorbunov, A. Gribushin, V. Ivanchenko, Y. Ivanov, V. Kachanov, L. Kardapoltsev, V. Karjavine, A. Karneyeu, V. Kim, M. Kirakosyan, D. Kirpichnikov, M. Kirsanov, V. Klyukhin, O. Kodolova, D. Konstantinov, V. Korenkov, A. Kozyrev, N. Krasnikov, E. Kuznetsova, A. Lanev, A. Litomin, O. Lukina, N. Lychkovskaya, V. Makarenko, A. Malakhov, V. Matveev, V. Murzin, A. Nikitenko, S. Obraztsov, V. Okhotnikov, V. Oreshkin, A. Oskin, I. Ovtin, V. Palichik, P. Parygin, A. Pashenkov, V. Perelygin, S. Petrushanko, G. Pivovarov, S. Polikarpov, V. Popov, O. Radchenko, M. Savina, V. Savrin, V. Shalaev, S. Shmatov, S. Shulha, Y. Skovpen, S. Slabospitskii, I. Smirnov, V. Smirnov, A. Snigirev, D. Sosnov, A. Stepennov, V. Sulimov, E. Tcherniaev, A. Terkulov, O. Teryaev, M. Toms, A. Toropin, L. Uvarov, A. Uzunian, E. Vlasov, S. Volkov, A. Vorobyev, N. Voytishin, B. S. Yuldashev, A. Zarubin, I. Zhizhin, A. Zhokin, CMS Collaboration

**Affiliations:** 1 Yerevan Physics Institute, Yerevan, Armenia; 2 Institut für Hochenergiephysik, Vienna, Austria; 3 Universiteit Antwerpen, Antwerpen, Belgium; 4 Vrije Universiteit Brussel, Brussel, Belgium; 5 Université Libre de Bruxelles, Bruxelles, Belgium; 6 Ghent University, Ghent, Belgium; 7 Université Catholique de Louvain, Louvain-la-Neuve, Belgium; 8 Centro Brasileiro de Pesquisas Fisicas, Rio de Janeiro, Brazil; 9 Universidade do Estado do Rio de Janeiro, Rio de Janeiro, Brazil; 10 Universidade Estadual Paulista, Universidade Federal do ABC, São Paulo, Brazil; 11 Institute for Nuclear Research and Nuclear Energy, Bulgarian Academy of Sciences, Sofia, Bulgaria; 12 University of Sofia, Sofia, Bulgaria; 13 Beihang University, Beijing, China; 14 Department of Physics, Tsinghua University, Beijing, China; 15 Institute of High Energy Physics, Beijing, China; 16 State Key Laboratory of Nuclear Physics and Technology, Peking University, Beijing, China; 17 Sun Yat-Sen University, Guangzhou, China; 18 Institute of Modern Physics and Key Laboratory of Nuclear Physics and Ion-beam Application (MOE), Fudan University, Shanghai, China; 19 Zhejiang University, Hangzhou, Zhejiang, China; 20 Universidad de Los Andes, Bogotá, Colombia; 21 Universidad de Antioquia, Medellin, Colombia; 22 Faculty of Electrical Engineering, Mechanical Engineering and Naval Architecture, University of Split, Split, Croatia; 23 Faculty of Science, University of Split, Split, Croatia; 24 Institute Rudjer Boskovic, Zagreb, Croatia; 25 University of Cyprus, Nicosia, Cyprus; 26 Charles University, Prague, Czech Republic; 27 Escuela Politecnica Nacional, Quito, Ecuador; 28 Universidad San Francisco de Quito, Quito, Ecuador; 29 Academy of Scientific Research and Technology of the Arab Republic of Egypt, Egyptian Network of High Energy Physics, Cairo, Egypt; 30 Center for High Energy Physics (CHEP-FU), Fayoum University, El-Fayoum, Egypt; 31 National Institute of Chemical Physics and Biophysics, Tallinn, Estonia; 32 Department of Physics, University of Helsinki, Helsinki, Finland; 33 Helsinki Institute of Physics, Helsinki, Finland; 34 Lappeenranta-Lahti University of Technology, Lappeenranta, Finland; 35 IRFU, CEA, Université Paris-Saclay, Gif-sur-Yvette, France; 36 Laboratoire Leprince-Ringuet, CNRS/IN2P3, Ecole Polytechnique, Institut Polytechnique de Paris, Palaiseau, France; 37 Université de Strasbourg, CNRS, IPHC UMR 7178, Strasbourg, France; 38 Institut de Physique des 2 Infinis de Lyon (IP2I ), Villeurbanne, France; 39 Georgian Technical University, Tbilisi, Georgia; 40 I. Physikalisches Institut, RWTH Aachen University, Aachen, Germany; 41 III. Physikalisches Institut A, RWTH Aachen University, Aachen, Germany; 42 III. Physikalisches Institut B, RWTH Aachen University, Aachen, Germany; 43 Deutsches Elektronen-Synchrotron, Hamburg, Germany; 44 University of Hamburg, Hamburg, Germany; 45 Karlsruher Institut fuer Technologie, Karlsruhe, Germany; 46 Institute of Nuclear and Particle Physics (INPP), NCSR Demokritos, Aghia Paraskevi, Greece; 47 National and Kapodistrian University of Athens, Athens, Greece; 48 National Technical University of Athens, Athens, Greece; 49 University of Ioánnina, Ioannina, Greece; 50 MTA-ELTE Lendület CMS Particle and Nuclear Physics Group, Eötvös Loránd University, Budapest, Hungary; 51 Wigner Research Centre for Physics, Budapest, Hungary; 52 Institute of Nuclear Research ATOMKI, Debrecen, Hungary; 53 Institute of Physics, University of Debrecen, Debrecen, Hungary; 54 Karoly Robert Campus, MATE Institute of Technology, Gyongyos, Hungary; 55 Panjab University, Chandigarh, India; 56 University of Delhi, Delhi, India; 57 Saha Institute of Nuclear Physics, HBNI, Kolkata, India; 58 Indian Institute of Technology Madras, Madras, India; 59 Bhabha Atomic Research Centre, Mumbai, India; 60 Tata Institute of Fundamental Research-A, Mumbai, India; 61 Tata Institute of Fundamental Research-B, Mumbai, India; 62 National Institute of Science Education and Research, An OCC of Homi Bhabha National Institute, Bhubaneswar, Odisha, India; 63 Indian Institute of Science Education and Research (IISER), Pune, India; 64 Isfahan University of Technology, Isfahan, Iran; 65 Institute for Research in Fundamental Sciences (IPM), Tehran, Iran; 66 University College Dublin, Dublin, Ireland; 67 INFN Sezione di Bari, Università di Bari, Politecnico di Bari, Bari, Italy; 68 INFN Sezione di Bologna, Università di Bologna, Bologna, Italy; 69 INFN Sezione di Catania, Università di Catania, Catania, Italy; 70 INFN Sezione di Firenze, Università di Firenze, Firenze, Italy; 71 INFN Laboratori Nazionali di Frascati, Frascati, Italy; 72 INFN Sezione di Genova, Università di Genova, Genoa, Italy; 73 INFN Sezione di Milano-Bicocca, Università di Milano-Bicocca, Milan, Italy; 74 INFN Sezione di Napoli, Università di Napoli ’Federico II’, Napoli, Italy; Università della Basilicata, Potenza, Italy; Università G. Marconi, Rome, Italy; 75 INFN Sezione di Padova, Università di Padova, Padova, Italy; Università di Trento, Trento, Italy; 76 INFN Sezione di Pavia, Università di Pavia, Pavia, Italy; 77 INFN Sezione di Perugia, Università di Perugia, Perugia, Italy; 78 INFN Sezione di Pisa, Università di Pisa, Scuola Normale Superiore di Pisa, Pisa, Italy; Università di Siena, Siena, Italy; 79 INFN Sezione di Roma, Sapienza Università di Roma, Rome, Italy; 80 INFN Sezione di Torino, Università di Torino, Torino, Italy; Università del Piemonte Orientale, Novara, Italy; 81 INFN Sezione di Trieste, Università di Trieste, Trieste, Italy; 82 Kyungpook National University, Daegu, Korea; 83 Chonnam National University, Institute for Universe and Elementary Particles, Kwangju, Korea; 84 Hanyang University, Seoul, Korea; 85 Korea University, Seoul, Korea; 86 Department of Physics, Kyung Hee University, Seoul, Korea; 87 Sejong University, Seoul, Korea; 88 Seoul National University, Seoul, Korea; 89 University of Seoul, Seoul, Korea; 90 Department of Physics, Yonsei University, Seoul, Korea; 91 Sungkyunkwan University, Suwon, Korea; 92 College of Engineering and Technology, American University of the Middle East (AUM), Dasman, Kuwait; 93 Riga Technical University, Riga, Latvia; 94 Vilnius University, Vilnius, Lithuania; 95 National Centre for Particle Physics, Universiti Malaya, Kuala Lumpur, Malaysia; 96 Universidad de Sonora (UNISON), Hermosillo, Mexico; 97 Centro de Investigacion y de Estudios Avanzados del IPN, Mexico City, Mexico; 98 Universidad Iberoamericana, Mexico City, Mexico; 99 Benemerita Universidad Autonoma de Puebla, Puebla, Mexico; 100 University of Montenegro, Podgorica, Montenegro; 101 University of Auckland, Auckland, New Zealand; 102 University of Canterbury, Christchurch, New Zealand; 103 National Centre for Physics, Quaid-I-Azam University, Islamabad, Pakistan; 104 AGH University of Science and Technology Faculty of Computer Science, Electronics and Telecommunications, Kraków, Poland; 105 National Centre for Nuclear Research, Swierk, Poland; 106 Institute of Experimental Physics, Faculty of Physics, University of Warsaw, Warsaw, Poland; 107 Laboratório de Instrumentação e Física Experimental de Partículas, Lisbon, Portugal; 108 VINCA Institute of Nuclear Sciences, University of Belgrade, Belgrade, Serbia; 109 Centro de Investigaciones Energéticas Medioambientales y Tecnológicas (CIEMAT), Madrid, Spain; 110 Universidad Autónoma de Madrid, Madrid, Spain; 111 Instituto Universitario de Ciencias y Tecnologías Espaciales de Asturias (ICTEA), Universidad de Oviedo, Oviedo, Spain; 112 Instituto de Física de Cantabria (IFCA), CSIC-Universidad de Cantabria, Santander, Spain; 113 University of Colombo, Colombo, Sri Lanka; 114 University of Ruhuna, Department of Physics, Matara, Sri Lanka; 115 CERN, European Organization for Nuclear Research, Geneva, Switzerland; 116 Paul Scherrer Institut, Villigen, Switzerland; 117 ETH Zurich-Institute for Particle Physics and Astrophysics (IPA), Zurich, Switzerland; 118 Universität Zürich, Zurich, Switzerland; 119 National Central University, Chung-Li, Taiwan; 120 National Taiwan University (NTU), Taipei, Taiwan; 121 Department of Physics, Faculty of Science, Chulalongkorn University, Bangkok, Thailand; 122 Physics Department, Science and Art Faculty, Çukurova University, Adana, Turkey; 123 Physics Department, Middle East Technical University, Ankara, Turkey; 124 Bogazici University, Istanbul, Turkey; 125 Istanbul Technical University, Istanbul, Turkey; 126 Istanbul University, Istanbul, Turkey; 127 Institute for Scintillation Materials of National Academy of Science of Ukraine, Kharkiv, Ukraine; 128 National Science Centre, Kharkiv Institute of Physics and Technology, Kharkiv, Ukraine; 129 University of Bristol, Bristol, UK; 130 Rutherford Appleton Laboratory, Didcot, UK; 131 Imperial College, London, UK; 132 Brunel University, Uxbridge, UK; 133 Baylor University, Waco, TX USA; 134 Catholic University of America, Washington, DC USA; 135 The University of Alabama, Tuscaloosa, AL USA; 136 Boston University, Boston, MA USA; 137 Brown University, Providence, RI USA; 138 University of California, Davis, Davis, CA USA; 139 University of California, Los Angeles, CA USA; 140 University of California, Riverside, Riverside, CA USA; 141 University of California, San Diego, La Jolla, CA USA; 142 Department of Physics, University of California, Santa Barbara, Santa Barbara, CA USA; 143 California Institute of Technology, Pasadena, CA USA; 144 Carnegie Mellon University, Pittsburgh, PA USA; 145 University of Colorado Boulder, Boulder, CO USA; 146 Cornell University, Ithaca, NY USA; 147 Fermi National Accelerator Laboratory, Batavia, IL USA; 148 University of Florida, Gainesville, FL USA; 149 Florida State University, Tallahassee, FL USA; 150 Florida Institute of Technology, Melbourne, FL USA; 151 University of Illinois at Chicago (UIC), Chicago, IL USA; 152 The University of Iowa, Iowa City, IA USA; 153 Johns Hopkins University, Baltimore, MD USA; 154 The University of Kansas, Lawrence, KS USA; 155 Kansas State University, Manhattan, KS USA; 156 Lawrence Livermore National Laboratory, Livermore, CA USA; 157 University of Maryland, College Park, MD USA; 158 Massachusetts Institute of Technology, Cambridge, MA USA; 159 University of Minnesota, Minneapolis, MN USA; 160 University of Nebraska-Lincoln, Lincoln, NE USA; 161 State University of New York at Buffalo, Buffalo, NY USA; 162 Northeastern University, Boston, MA USA; 163 Northwestern University, Evanston, IL USA; 164 University of Notre Dame, Notre Dame, IN USA; 165 The Ohio State University, Columbus, OH USA; 166 Princeton University, Princeton, NJ USA; 167 University of Puerto Rico, Mayaguez, PR USA; 168 Purdue University, West Lafayette, IN USA; 169 Purdue University Northwest, Hammond, IN USA; 170 Rice University, Houston, TX USA; 171 University of Rochester, Rochester, NY USA; 172 The Rockefeller University, New York, NY USA; 173 Rutgers, The State University of New Jersey, Piscataway, NJ USA; 174 University of Tennessee, Knoxville, TN USA; 175 Texas A &M University, College Station, TX USA; 176 Texas Tech University, Lubbock, TX USA; 177 Vanderbilt University, Nashville, TN USA; 178 University of Virginia, Charlottesville, VA USA; 179 Wayne State University, Detroit, MI USA; 180 University of Wisconsin-Madison, Madison, WI USA; 181 Authors affiliated with an institute or an international laboratory covered by a cooperation agreement with CERN, Geneva, Switzerland; 182 Yerevan State University, Yerevan, Armenia; 183 TU Wien, Vienna, Austria; 184 Institute of Basic and Applied Sciences, Faculty of Engineering, Arab Academy for Science, Technology and Maritime Transport, Alexandria, Egypt; 185 Université Libre de Bruxelles, Bruxelles, Belgium; 186 Universidade Estadual de Campinas, Campinas, Brazil; 187 Federal University of Rio Grande do Sul, Porto Alegre, Brazil; 188 UFMS, Nova Andradina, Brazil; 189 The University of the State of Amazonas, Manaus, Brazil; 190 University of Chinese Academy of Sciences, Beijing, China; 191 Nanjing Normal University Department of Physics, Nanjing, China; 192 The University of Iowa, Iowa City, IA USA; 193 University of Chinese Academy of Sciences, Beijing, China; 194 an institute or an international laboratory covered by a cooperation agreement with CERN, Geneva, Switzerland; 195 Ain Shams University, Cairo, Egypt; 196 British University in Egypt, Cairo, Egypt; 197 Purdue University, West Lafayette, IN USA; 198 Université de Haute Alsace, Mulhouse, France; 199 Ilia State University, Tbilisi, Georgia; 200 Erzincan Binali Yildirim University, Erzincan, Turkey; 201 CERN, European Organization for Nuclear Research, Geneva, Switzerland; 202 University of Hamburg, Hamburg, Germany; 203 RWTH Aachen University, III. Physikalisches Institut A, Aachen, Germany; 204 Isfahan University of Technology, Isfahan, Iran; 205 Brandenburg University of Technology, Cottbus, Germany; 206 Forschungszentrum Jülich, Juelich, Germany; 207 Physics Department, Faculty of Science, Assiut University, Assiut, Egypt; 208 Karoly Robert Campus, MATE Institute of Technology, Gyongyos, Hungary; 209 Institute of Physics, University of Debrecen, Debrecen, Hungary; 210 Institute of Nuclear Research ATOMKI, Debrecen, Hungary; 211 Universitatea Babes-Bolyai-Facultatea de Fizica, Cluj-Napoca, Romania; 212 MTA-ELTE Lendület CMS Particle and Nuclear Physics Group, Eötvös Loránd University, Budapest, Hungary; 213 Faculty of Informatics, University of Debrecen, Debrecen, Hungary; 214 Wigner Research Centre for Physics, Budapest, Hungary; 215 Punjab Agricultural University, Ludhiana, India; 216 UPES-University of Petroleum and Energy Studies, Dehradun, India; 217 Shoolini University, Solan, India; 218 University of Hyderabad, Hyderabad, India; 219 University of Visva-Bharati, Santiniketan, India; 220 Indian Institute of Science (IISc), Bangalore, India; 221 Indian Institute of Technology (IIT), Mumbai, India; 222 IIT Bhubaneswar, Bhubaneswar, India; 223 Institute of Physics, Bhubaneswar, India; 224 Deutsches Elektronen-Synchrotron, Hamburg, Germany; 225 Department of Physics, Isfahan University of Technology, Isfahan, Iran; 226 Sharif University of Technology, Tehran, Iran; 227 Department of Physics, University of Science and Technology of Mazandaran, Behshahr, Iran; 228 Italian National Agency for New Technologies, Energy and Sustainable Economic Development, Bologna, Italy; 229 Centro Siciliano di Fisica Nucleare e di Struttura Della Materia, Catania, Italy; 230 Scuola Superiore Meridionale, Università di Napoli ’Federico II’, Naples, Italy; 231 Università di Napoli ’Federico II’, Naples, Italy; 232 Consiglio Nazionale delle Ricerche-Istituto Officina dei Materiali, Perugia, Italy; 233 Department of Applied Physics, Faculty of Science and Technology, Universiti Kebangsaan Malaysia, Bangi, Malaysia; 234 Consejo Nacional de Ciencia y Tecnología, Mexico City, Mexico; 235 IRFU, CEA, Université Paris-Saclay, Gif-sur-Yvette, France; 236 Faculty of Physics, University of Belgrade, Belgrade, Serbia; 237 Trincomalee Campus, Eastern University, Sri Lanka, Nilaveli, Sri Lanka; 238 INFN Sezione di Pavia, Università di Pavia, Pavia, Italy; 239 National and Kapodistrian University of Athens, Athens, Greece; 240 Ecole Polytechnique Fédérale Lausanne, Lausanne, Switzerland; 241 Universität Zürich, Zurich, Switzerland; 242 Stefan Meyer Institute for Subatomic Physics, Vienna, Austria; 243 Laboratoire d’Annecy-le-Vieux de Physique des Particules, IN2P3-CNRS, Annecy-le-Vieux, France; 244 Şırnak University, Sirnak, Turkey; 245 Near East University, Research Center of Experimental Health Science, Mersin, Turkey; 246 Konya Technical University, Konya, Turkey; 247 Izmir Bakircay University, Izmir, Turkey; 248 Adiyaman University, Adiyaman, Turkey; 249 Necmettin Erbakan University, Konya, Turkey; 250 Bozok Universitetesi Rektörlügü, Yozgat, Turkey; 251 Marmara University, Istanbul, Turkey; 252 Milli Savunma University, Istanbul, Turkey; 253 Kafkas University, Kars, Turkey; 254 Istanbul Bilgi University, Istanbul, Turkey; 255 Hacettepe University, Ankara, Turkey; 256 Faculty of Engineering, Istanbul University-Cerrahpasa, Istanbul, Turkey; 257 Yildiz Technical University, Istanbul, Turkey; 258 Vrije Universiteit Brussel, Brussel, Belgium; 259 School of Physics and Astronomy, University of Southampton, Southampton, UK; 260 IPPP Durham University, Durham, UK; 261 Monash University, Faculty of Science, Clayton, Australia; 262 Università di Torino, Turin, Italy; 263 Bethel University, St. Paul, MN USA; 264 Karamanoğlu Mehmetbey University, Karaman, Turkey; 265 California Institute of Technology, Pasadena, CA USA; 266 United States Naval Academy, Annapolis, MD USA; 267 Bingol University, Bingol, Turkey; 268 Georgian Technical University, Tbilisi, Georgia; 269 Sinop University, Sinop, Turkey; 270 Erciyes University, Kayseri, Turkey; 271 Institute of Modern Physics and Key Laboratory of Nuclear Physics and Ion-beam Application (MOE)-Fudan University, Shanghai, China; 272 Texas A &M University at Qatar, Doha, Qatar; 273 Kyungpook National University, Daegu, Korea; 274 another institute or international laboratory covered by a cooperation agreement with CERN, Geneva, Switzerland; 275 Yerevan Physics Institute, Yerevan, Armenia; 276 University of Florida, Gainesville, FL USA; 277 Imperial College, London, UK; 278 Institute of Nuclear Physics of the Uzbekistan Academy of Sciences, Tashkent, Uzbekistan; 279 CERN, 1211 Geneva 23, Switzerland

## Abstract

The production of Z bosons associated with jets is measured in $\text {p}\text {p}$ collisions at $\sqrt{s}=13\,\text {Te}\hspace{-.08em}\text {V}$ with data recorded with the CMS experiment at the LHC corresponding to an integrated luminosity of 36.3$\,\text {fb}^{-1}$. The multiplicity of jets with transverse momentum $p_{\textrm{T}} > 30\,\text {Ge}\hspace{-.08em}\text {V}$ is measured for different regions of the Z boson’s $p_{\textrm{T}} (\text {Z })$, from lower than 10$\,\text {Ge}\hspace{-.08em}\text {V}$ to higher than 100$\,\text {Ge}\hspace{-.08em}\text {V}$. The azimuthal correlation $\varDelta \phi$ between the Z boson and the leading jet, as well as the correlations between the two leading jets are measured in three regions of $p_{\textrm{T}} (\text {Z })$. The measurements are compared with several predictions at leading and next-to-leading orders, interfaced with parton showers. Predictions based on transverse-momentum dependent parton distributions and corresponding parton showers give a good description of the measurement in the regions where multiple parton interactions and higher jet multiplicities are not important. The effects of multiple parton interactions are shown to be important to correctly describe the measured spectra in the low $p_{\textrm{T}} (\text {Z })$ regions.

## Introduction

In high-energy proton–proton ($\text {p}\text {p}$) collisions at the CERN LHC, the production of Z bosons is regarded as a standard measurement tool, because their properties can be measured very precisely in their leptonic decay channel, and the production cross section can be calculated with high precision. Although the production of Z bosons is a purely electroweak (EW) process, corrections from quantum chromodynamics (QCD) play an increasingly important role as the Z boson transverse momentum $p_{\textrm{T}} (\text {Z })$ increases. At small $p_{\textrm{T}} (\text {Z })$, where soft-gluon radiation is important, a resummation to all orders must be performed in order to obtain stable theoretical predictions [1–4] and to describe the measurements [5]. When $p_{\textrm{T}} (\text {Z })$ increases, hard partonic radiation becomes important and associated jets can be measured, allowing the study of QCD contributions to Z production.

Cross sections for the production of Z bosons associated with jets were measured in proton–antiproton collisions at $\sqrt{s}=1.96\,\text {Te}\hspace{-.08em}\text {V}$ at the Fermilab Tevatron by the CDF and D0 Collaborations [6, 7]. At the LHC, the ATLAS, CMS, and LHCb Collaborations have published measurements in $\text {p}\text {p}$ collisions at $\sqrt{s}=7\,\text {Te}\hspace{-.08em}\text {V}$ [8–13], 8$\,\text {Te}\hspace{-.08em}\text {V}$  [14, 15], and 13$\,\text {Te}\hspace{-.08em}\text {V}$  [16, 17].

This article describes a study by the CMS Collaboration of the production of Z bosons with associated jets at a center-of-mass energy of 13$\,\text {Te}\hspace{-.08em}\text {V}$. We measure the multiplicity of jets with $p_{\textrm{T}} > 30\,\text {Ge}\hspace{-.08em}\text {V}$ in a pseudorapidity range of $|\eta |<2.4$. In the region of low $p_{\textrm{T}} (\text {Z })$, additional jets must balance the leading jet of $p_{\textrm{T}} > 30\,\text {Ge}\hspace{-.08em}\text {V}$, whereas at large $p_{\textrm{T}} (\text {Z })$ the Z boson is expected to balance the $p_{\textrm{T}}$ of the leading jet. We measure distributions in three (representative) $p_{\textrm{T}} (\text {Z })$ regions: at low transverse momentum $p_{\textrm{T}} (\text {Z }) < 10\,\text {Ge}\hspace{-.08em}\text {V}$; in the intermediate range of $30< p_{\textrm{T}} (\text {Z }) < 50\,\text {Ge}\hspace{-.08em}\text {V}$; and in the large range of $p_{\textrm{T}} (\text {Z }) > 100\,\text {Ge}\hspace{-.08em}\text {V}$.

The jet multiplicity, the azimuthal correlation $\varDelta \phi (\text {Z }j_1)$ between the Z boson and the leading jet, as well as the correlation $\varDelta \phi (j_1j_2)$ between the two leading jets, is measured in these three ranges of $p_{\textrm{T}} (\text {Z })$. At small $p_{\textrm{T}} (\text {Z })$, a weak correlation between the Z boson and the leading jet is expected, whereas at large $p_{\textrm{T}} (\text {Z })$ the azimuthal correlation is expected to be strong, since then the Z boson and the leading jet are most likely the highest $p_{\textrm{T}}$ objects in the event. The situation is opposite for $\varDelta \phi (j_1j_2)$, where at small $p_{\textrm{T}} (\text {Z })$ a strong correlation is expected, whereas at large $p_{\textrm{T}} (\text {Z })$ the correlation will be weak.

The measurement of jet multiplicity as well as the measurements of the azimuthal correlations $\varDelta \phi (\text {Z }j_1)$, and $\varDelta \phi (j_1j_2)$ in various ranges of $p_{\textrm{T}} (\text {Z })$ provide an opportunity to make detailed comparisons with theoretical predictions. In particular, calculations of next-to-leading order (NLO) Z +jet production supplemented with parton shower (PS) and hadronization, as well as merged calculations with higher partonic jet multiplicity, can be studied. Of particular interest are the comparisons with predictions based on the parton branching (PB) method with transverse-momentum dependent (PB-TMD) parton distribution functions (PDFs) [18–20] together with a TMD-based PS [21]. A comparison with resummed calculations using the Geneva [22–25] framework is also shown.

## The CMS detector

The central feature of the CMS apparatus is a superconducting solenoid of 6$\,\text {m}$ internal diameter, providing a magnetic field of 3.8$\,\text {T}$. Within the solenoid volume are a silicon pixel and strip tracker, a lead tungstate crystal electromagnetic calorimeter (ECAL), and a brass and scintillator hadron calorimeter (HCAL), each composed of a barrel and two endcap sections. Forward calorimeters extend the $\eta$ coverage provided by the barrel and endcap detectors. Muons are detected in gas-ionization chambers embedded in the steel flux-return yoke outside the solenoid.

Events of interest are selected using a two-tiered trigger system. The first level, composed of custom hardware processors, uses information from the calorimeters and muon detectors to select events at a rate of around 100$\,\text {kHz}$ within a fixed latency of about 4 $\upmu$s [26]. The second level, known as the high-level trigger, consists of a farm of processors running a version of the full event reconstruction software optimized for fast processing and reduces the event rate to around 1$\,\text {kHz}$ before data storage [27].

The particle-flow algorithm (PF) [28] reconstructs and identifies each individual particle in an event, with an optimized combination of information from the various elements of the CMS detector. The primary vertex (PV) is taken to be the vertex corresponding to the hardest scattering in the event, evaluated using tracking information alone, as described in Section 9.4.1 of Ref. [29].

The energy of photons is obtained from the ECAL measurement. The energy of electrons is determined from a combination of the electron momentum at the primary interaction vertex as determined by the tracker, the energy of the corresponding ECAL cluster, and the energy sum of all bremsstrahlung photons spatially compatible with originating from the electron track. The momentum resolution for electrons with $p_{\textrm{T}} \approx 45\,\text {Ge}\hspace{-.08em}\text {V}$ from $\text {Z }\rightarrow \hbox {e}^{+}\hbox {e}^{-}$ decays ranges from 1.7 to 4.5%. It is generally better in the barrel region than in the endcaps, and also depends on the bremsstrahlung energy emitted by the electron as it traverses the material in front of the ECAL [30, 31]. The overall reconstruction efficiency is around 93% for electrons from Z decay.

The energy of muons is obtained from the curvature of the corresponding track. Muons are measured in the range $|\eta |<2.4$, with detection planes made using three technologies: drift tubes, cathode strip chambers, and resistive-plate chambers. The single-muon trigger efficiency exceeds 90% over the full $\eta$ range, and the efficiency to reconstruct and identify muons is greater than 96%. Matching muons to tracks measured in the silicon tracker results in a relative $p_{\textrm{T}}$ resolution of 1% in the barrel and 3% in the endcaps for muons with $p_{\textrm{T}}$ up to 100$\,\text {Ge}\hspace{-.08em}\text {V}$. The $p_{\textrm{T}}$ resolution in the barrel is better than 7% for muons with $p_{\textrm{T}}$ up to 1$\,\text {Te}\hspace{-.08em}\text {V}$  [32].

The energy of charged hadrons is determined from a combination of their momentum measured in the tracker and the matching ECAL and HCAL energy deposits, corrected for the response function of the calorimeters to hadronic showers. Finally, the energy of neutral hadrons is obtained from the corresponding corrected ECAL and HCAL energies.

For each event, hadronic jets are clustered from these reconstructed particles using the infrared and collinear safe anti-$k_{\textrm{T}}$ algorithm [33, 34] with a distance parameter of 0.4. Jet momentum is determined as the vectorial sum of all particle momenta in the jet and is found from simulation to be, on average, within 5–10% of the true momentum over the whole $p_{\textrm{T}}$ spectrum and detector acceptance. Additional $\text {p}\text {p}$ interactions within the same or nearby bunch crossings (pileup) contribute additional tracks and calorimetric energy depositions, increasing the apparent jet momentum. To mitigate this effect, tracks identified as originating from pileup vertices are discarded and a correction is applied to correct for any remaining contributions.

Jet energy corrections are derived from simulation studies so the average measured energy of jets becomes identical to that of particle level jets. In situ measurements of the momentum balance in dijet, photon+jet, Z +jet, and multijet events are used to determine any residual differences between the jet energy scale in data and in simulation, and appropriate corrections are made [35]. Additional selection criteria are applied to each jet to remove jets potentially dominated by instrumental effects or reconstruction failures. The jet energy resolution amounts typically to 15–20% at 30$\,\text {Ge}\hspace{-.08em}\text {V}$, 10% at 100$\,\text {Ge}\hspace{-.08em}\text {V}$, and 5% at 1$\,\text {Te}\hspace{-.08em}\text {V}$  [35].

During the 2016 data-taking, a gradual shift in the timing of the inputs of the ECAL first-level trigger in the region at $|\eta | > 2.0$, referred to as prefiring, caused a specific trigger inefficiency. For events containing an electron (a jet) with $p_{\textrm{T}}$ larger than 50 (100)$\,\text {Ge}\hspace{-.08em}\text {V}$, in the region $2.5< |\eta | < 3.0$ the efficiency loss is about 10–20%, depending on $p_{\textrm{T}}$, $\eta$, and timing. Correction factors were computed from data and applied to the acceptance evaluated by simulation.

A more detailed description of the CMS detector, together with a definition of the coordinate system used and the relevant kinematic variables, is reported in Ref. [36].

## Theoretical predictions

The measured differential cross sections are compared with a variety of predictions. One of the NLO calculations uses MadGraph 5_amc@nlo  [37] (version 2.2.2) event generator interfaced with pythia 8  [38] for PS and hadronization. The matrix element calculations include $\text {Z }/\gamma ^{*}$+0,1,2 jets at NLO. It is labeled as MG5_aMC+Py8 ($\le 2j$ NLO) in the following.

The measurements are also compared with predictions obtained from MadGraph 5_amc@nlo (version 2.6.9) with PB-TMD PDFs and the corresponding PS as implemented in Cascade3  [21] (labeled as MG5_aMC+CA3). The matrix elements (MEs) are calculated at NLO for Z +1 and Z +2 partons separately. The parton density PB-NLO-HERAI$+$II-2018-set2 [20], as well as the PB initial-state PS, follow angular ordering conditions [39–42]. The advantage of the MG5_aMC+CA3 calculation is that the parameters of the PB-TMD initial-state PS are fixed by the PB-TMD PDFs.

In all calculations using MadGraph 5_amc@nlo the renormalization and factorization scales are set to $\mu _\textrm{r}=\mu _\textrm{f}=1/2 \sum _i \textrm{H}_{\textrm{T}\,i}$, where $\textrm{H}_{\textrm{T}\,i}$ is the scalar sum of the $p_{\textrm{T}}$ with *i* running over all final particles and partons in the ME calculation. The corresponding uncertainties are estimated as the envelope of the set of variations of $\mu _\textrm{r}$ and $\mu _\textrm{f}$ by factors of 2 and 1/2, in all possible combinations except the extreme cases $(\mu _\textrm{f}, \mu _\textrm{r})=(2,0.5), (0.5,2)$. The PDF uncertainties are estimated as the standard deviation of observables when using weights from the replicas provided in the NNPDF 3.0 NLO [43] PDF set.

The corresponding versions of these generators at leading-order (LO) are also compared with the measurement.

The following calculations are used for comparison with the measurements (a summary is given in Table 1):

- MG5_aMC+Py8 ($\le 2j$ NLO) is a fixed-order perturbative QCD calculation at NLO of up to 2 noncollinear high-$p_{\textrm{T}}$ partons for $\text {p}\text {p}\rightarrow \text {Z }$+*N*, $N=0$, 1, 2, supplemented with PS and multiparton interactions (MPIs) from pythia 8 (version 8.212). The parameters of the underlying event tune CUETP8M1 [44] are applied. The merging of PS and MEs is performed with the FxFx scheme [45] with the merging scale of 30$\,\text {Ge}\hspace{-.08em}\text {V}$ and a minimal partonic $p_{\textrm{T}}$ for jets of $p_{\textrm{T}} ^{\text {part}} = 15\,\text {Ge}\hspace{-.08em}\text {V}$. The NNPDF 3.0 NLO PDFs are used and $\alpha _\textrm{S} (m_\text {Z }) =0.118$ is chosen, where $m_\text {Z }$ is the Z boson mass. The predictions from MG5_aMC+Py8 ($\le 2j$ NLO) are used to investigate the effect of MPI.
- MG5_aMC+Py8 ($\le 4j$ LO) includes MEs computed at LO for $\text {p}\text {p}\rightarrow \text {Z }$+*N* partons, $N=0,1\ldots 4$, using the $k_{\textrm{T}}$-MLM [46] procedure to match the different parton multiplicities of the MEs to the PS, with the matching scale set to 19$\,\text {Ge}\hspace{-.08em}\text {V}$. The pythia 8 generator (version 8.212) is interfaced with MG5_aMC to include initial- and final-state PS and hadronization, with settings defined by the CUETP8M1 tune [44]. The NNPDF 2.3 LO [47, 48] PDF is used, and the strong coupling $\alpha _\textrm{S} (m_\text {Z })$ is set to 0.130. The total cross section for $\text {Z }{}\rightarrow \ell ^+ \ell ^- + \ge$0 jet is normalized to the predictions of fewz v3.1 next-to-next-to-leading order (NNLO) [49] applying a K-factor of 1.17.
- MG5_aMC+CA3 (Z +1 NLO) is a fixed-order perturbative QCD calculation at NLO of one noncollinear high-$p_{\textrm{T}}$ parton for $\text {p}\text {p}\rightarrow \text {Z }$+1 with $p_{\textrm{T}} ^\text {part}>15\,\text {Ge}\hspace{-.08em}\text {V}$, supplemented with PB-TMD PDFs and PS, which for the initial state follows the PB-TMD distribution [21]. The NLO PB-TMD set 2 [20] with $\alpha _\textrm{S} (m_\text {Z })= 0.118$ is used, the collinear version of PB set 2 is used for the ME calculation. This leads to cross sections 10–20% smaller than obtained with other PDFs because PB parton densities are determined from a fit to HERA data only. Therefore, an overall normalization factor of 1.2 is applied to the PB prediction. The inclusion of the transverse momentum $k_{\textrm{T}}$ and initial-state PS is performed with Cascade3 [21] (version 3.2.1). Final-state radiation, which is not constrained by the PB-TMD PDF, and hadronization is performed with pythia 6 (version 6.428) [50]. MPI effects are not simulated in this approach.
- MG5_aMC+CA3 (Z +2 NLO) is a fixed-order perturbative QCD calculation at NLO of two noncollinear high-$p_{\textrm{T}}$ partons for $\text {p}\text {p}\rightarrow \text {Z }$+2 with $p_{\textrm{T}} ^\text {part}>15\,\text {Ge}\hspace{-.08em}\text {V}$, supplemented with PB-TMD PDFs and parton showering and hadronization. The same PB-TMD distribution and PS as in MG5_aMC+CA3 (Z +1 NLO) is applied.
- MG5_aMC+CA3 (Z $\le 3j$ LO) uses MG5_aMC to generate $\text {Z }$+0,1,2,3 jet samples at LO with a partonic generation cut $p_{\textrm{T}} ^\text {part}> 15\,\text {Ge}\hspace{-.08em}\text {V}$. The TMD merging [51] procedure for combining the TMD PS with the ME calculations is used. The same PB-TMD distributions and PS as in MG5_aMC+CA3 (Z +1 NLO) are applied. A merging scale value of 23$\,\text {Ge}\hspace{-.08em}\text {V}$ is used, since it provides a smooth transition between ME and PS computations. An overall K-factor of 1.27 is applied to the prediction. MPI effects are not simulated.
- Geneva (Z +0 NNLO) (1.0-RC3) [22–25] is based on NNLO calculations for the processes $\text {p}\text {p}\rightarrow \text {Z }{}/\gamma \rightarrow \hbox {e}^{+}\hbox {e}^{-}$ and $\upmu ^{+}\upmu ^{-}$ combined with higher-order resummation. The calculation uses the PDF4LHC15 NNLO PDF set [52] with $\alpha _\textrm{S} (m_\text {Z })= 0.118$. The simulation of PS, hadronization and MPI is performed by pythia 8 (version 8.212) with the CUETP8M1 tune.

**Table 1:** Description of the simulated samples used in the analysis

Generator	PDF	Matrix element	Tune
MG5_aMC+Py8 ($\le 2j$ NLO) [37]	$\mathrm {NNPDF3.0}$ (NLO) [43]	NLO ($2\rightarrow \text {Z }{}$+0,1,2)	$\textrm{CUETP8M1}$ [44]
MG5_aMC+Py8 ($\le 4j$ LO) [37, 53]	$\mathrm {NNPDF2.3}$ (LO) [48]	LO ($2\rightarrow \text {Z }$+0,1,2,3,4)	$\textrm{CUETP8M1}$ [44]
MG5_aMC+CA3 (Z +1 NLO) [37]	${\textrm{PB}}\,{\textrm{NLO}}\,{\textrm{set2}}$ (NLO) [20]	NLO ($2\rightarrow \text {Z }$+1)	–
MG5_aMC+CA3 (Z +2 NLO) [37]	${\mathrm{PB~NLO~set2}}$ (NLO) [20]	NLO ($2\rightarrow \text {Z }$+2)	–
MG5_aMC+CA3 (Z $\le 3j$ LO) [37, 51]	${\mathrm{PB~NLO~set2}}$ (NLO) [20]	LO ($2\rightarrow \text {Z }$+0,1,2,3)	–
Geneva (Z +0 NNLO) [22–25]	$\mathrm {NNPDF3.1}$ (NLO) [54]	NNLO ($2\rightarrow \text {Z }$)	$\textrm{CUETP8M1}$ [44]

### Simulated samples

Events generated by MG5_aMC+Py8 ($\le 2j$ NLO) are passed through a full detector simulation based on Geant4  [55]. The simulated events are reconstructed using standard CMS reconstruction packages. This sample is used for the simulation of the signal process to estimate efficiencies, systematic uncertainties and for the correction of the data for detector spreading effects and inefficiencies, the so-called unfolding procedure.

Other processes that can give a final state with two oppositely charged same-flavor leptons and jets are ${\text {t}{}\overline{\text {t}}}$, single top, vector boson pair (VV) and W+jets. The ${\text {t}{}\overline{\text {t}}}$ and single top backgrounds are generated using POWHEG 2.0  [56–61] interfaced with pythia 8. The total cross section of ${\text {t}{}\overline{\text {t}}}$ production is normalized to the prediction with NNLO accuracy in QCD and next-to-next-to-leading logarithmic (NNLL) gluon radiation resummation calculated with Top$++$ 2.0 [62]. The double vector boson productions are generated with MG5_aMC  ($\text {W}\text {Z }$), Powheg ($\text {W}\text {W}$), both interfaced to pythia 8, or with pythia 8 for $\text {Z }\text {Z }$. The total cross sections for the $\text {W}\text {Z }$ and $\text {Z }\text {Z }$ diboson samples are normalized to the NLO prediction calculated with MCFM 6.6 [63]. The W+jets sample is generated by MG5_aMC at NLO accuracy, interfaced with pythia 8. The Z boson decay into $\uptau ^{+}\uptau ^{-}$ is included in the signal simulation and considered as a background.

## Data analysis

The differential cross section of Z bosons with associated jets is measured in bins of $p_{\textrm{T}} (\text {Z })$, as functions of the jet multiplicity, the azimuthal angles $\varDelta \phi (\text {Z }j_1)$ and $\varDelta \phi (j_1j_2)$, where $j_1$ is the leading jet and $j_2$ is the second-leading jet.

### Event selection

The data samples recorded in 2016 correspond to an integrated luminosity of 36.3$\,\text {fb}^{-1}$. Events with a pair of leptons ($\upmu ^{+}\upmu ^{-}$ or $\hbox {e}^{+}\hbox {e}^{-}$) consistent with the decay of a Z boson and with jets reconstructed from PF candidates are selected as Z +jet events. Those events are required to pass a series of selection criteria to reduce the background contributions. An event is selected if the double muon (electron) trigger with 18 and 7 (23 and 12)$\,\text {Ge}\hspace{-.08em}\text {V}$ thresholds in $p_{\textrm{T}}$ or a single muon trigger with a threshold of 24$\,\text {Ge}\hspace{-.08em}\text {V}$ is satisfied. In the offline selection, the leading (subleading) electron and muon candidates must have transverse momenta of $p_{\textrm{T}} > 25\,(20)\,\text {Ge}\hspace{-.08em}\text {V}$ in a range of $|\eta | < 2.4$. Only events with pairs of oppositely charged muons (electrons) with an invariant mass in the range $91\pm 15\,\text {Ge}\hspace{-.08em}\text {V}$ are accepted.

Muon candidates are required to be isolated from other particles, as specified by an isolation criteria, $I_\textrm{ISO}$:

$$\begin{aligned} I_\textrm{ISO}{} & {} =\left[ \sum ^\text {charged} p_{\textrm{T}} + \max \Big (0, \sum ^\text {neutral} p_{\textrm{T}} + \sum ^\textrm{EM} p_{\textrm{T}} \right. \\{} & {} \left. -0.5 \sum ^\textrm{PU} p_{\textrm{T}} \Big ) \right] /p_{\textrm{T}} ^{\upmu } \le 0.15, \end{aligned}$$

where the sums run over the corresponding particles inside a cone of radius $\varDelta R=\sqrt{\smash [b]{(\varDelta \eta )^2+(\varDelta \phi )^2}}=0.4$ around the muon candidate considering separately charged hadrons ($\text {charged}$), neutral hadrons ($\text {neutral}$), photons ($\textrm{EM}$), and charged particles from pileup ($\textrm{PU}$).

Electrons are required to be isolated from other particles, as specified by an isolation criteria, $I_\textrm{ISO}$:

$$\begin{aligned} I_\textrm{ISO}= & {} \left[ \sum ^\text {charged} p_{\textrm{T}} + \max \Big (0, \sum ^\text {neutral} p_{\textrm{T}} + \sum ^\textrm{EM} p_{\textrm{T}} \right. \\{} & {} \left. -\rho \textrm{A}_\text {eff}\Big ) \right] /p_{\textrm{T}} ^{\textrm{e}} \le 0.15, \end{aligned}$$

where the sums run over the corresponding particles inside a cone of radius $\varDelta R = 0.3$. The term $\rho \textrm{A}_\text {eff}$ represents a correction for pileup effects, where $\rho$ corresponds to the amount of $p_{\textrm{T}}$ added to the event per unit area and $\textrm{A}_\text {eff}$ is the area of the isolation region weighted by a factor that accounts for the dependence of the pileup transverse energy density on the electron $\eta$ [31, 64].

Jets are required to have a minimum $p_{\textrm{T}}$ of 30$\,\text {Ge}\hspace{-.08em}\text {V}$ to ensure that they are well measured and to reduce the pileup contamination. Jets are limited to a rapidity range of $|y |<2.4$, and are required to be isolated from the lepton candidates by $\varDelta R_{\ell ,j}>0.4$. To keep only charged particles originating from the Z boson vertex, charged particles identified as originating from pileup vertices are discarded. As discussed in Sect. 2, jet energy corrections are applied to data and simulation. The jet energy resolution (JER) in simulation is further spread to match that in data.

The simulated events are reweighted such that their pileup distribution matches the measured one in each data-taking period.

**Fig. 1:**
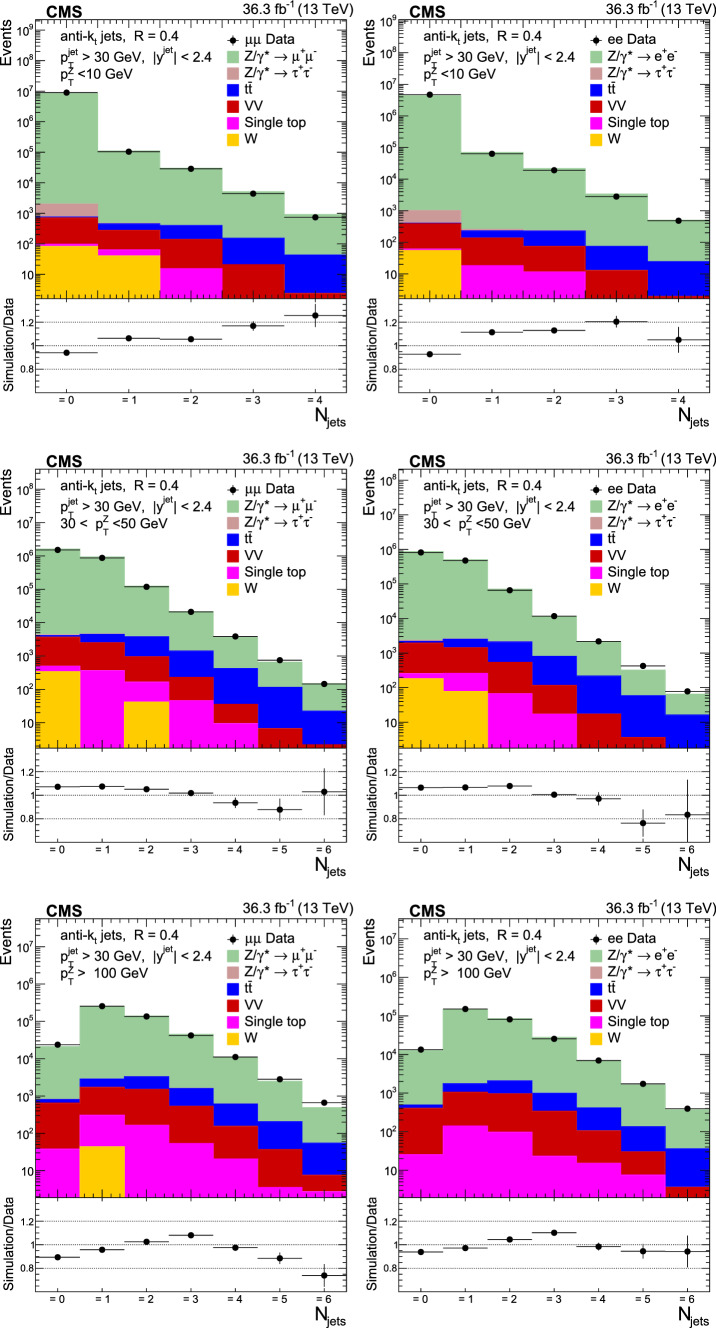
The exclusive jet multiplicity distribution before unfolding in three different regions of $p_{\textrm{T}} (\text {Z })$: $p_{\textrm{T}} (\text {Z }) <10\,\text {Ge}\hspace{-.08em}\text {V}$ (upper), $30<p_{\textrm{T}} (\text {Z }) <50\,\text {Ge}\hspace{-.08em}\text {V}$ (middle), $p_{\textrm{T}} (\text {Z }) >100\,\text {Ge}\hspace{-.08em}\text {V}$ (lower) for the $\upmu ^{+}$ $\upmu ^{-}$ channel (left) and the $\hbox {e}^{+}\hbox {e}^{-}$ channel (right). The error bars around the data points represent the statistical uncertainties

Several corrections for leptons are applied to the simulation yields to compensate for the measured differences between the efficiencies in data and simulation. These corrections are applied as trigger, lepton identification, and lepton isolation scale factors. The values of the scale factors are close to one. An additional trigger inefficiency correction due to the prefiring effect is included. The exclusive jet multiplicity in different regions of $p_{\textrm{T}} (\text {Z })$ for muon and electron channels is shown in Fig. 1.

### Correction for the detector effects

Detector effects, like inefficiencies and the spreading of the particle momentum, energy and angle, are corrected using the an unfolding procedure, which is applied after background subtraction. The iterative D’Agostini method as implemented in RooUnfold [65, 66] is used. The iteration is affected by fluctuations that increase with the number of iterations. The fluctuations are studied for each distribution and the procedure of unfolding is stopped before the fluctuations become significant with respect to the statistical uncertainty, following the method used in [16]. Through the unfolding procedure the cross section at the stable-particle level is obtained. Particles are considered stable if their proper lifetime is above 10$\,\text {mm}$/c. Neutrinos are not included. The momentum of the leptons is calculated including photons in a cone of a radius of $\varDelta R=0.1$ (“dressed” leptons). The phase space definition for the final cross sections is given in Table 2.

### Background estimation

The contributions from background processes are estimated using MC-based simulations, described in Sect. 3.1, and are subtracted from the measured distributions. The dominant background, $\text {t}\overline{\text {t}}$, is verified with data control samples, using the same criteria as for the measurement, but requiring the two leptons to have different flavours ($\textrm{e}\upmu$ instead of $\upmu ^{+}\upmu ^{-}$ or $\hbox {e}^{+}\hbox {e}^{-}$). The effect of mismodeling of top quark distributions is covered by the MC uncertainties. Therefore, no additional correction or uncertainty is applied [14]. The $\text {Z }{}\rightarrow \uptau ^{+}\uptau ^{-}$ decays are considered as a background, and their contribution is estimated from simulation and subtracted during the unfolding procedure.

### Uncertainties

The statistical uncertainties from the measured observables are propagated to the final results via the unfolding procedure. The systematic uncertainties originate from the following sources:

- Jet energy scale:
  Variations of the jet energy scale corrections [35, 67] are applied as functions of $p_{\textrm{T}}$ and $\eta$ for individual run periods; this affects the differential cross sections by 3–7%.
- Jet energy resolution:
  The JER [35, 67] uncertainty is obtained by varying the spreading factor to match the simulated jet energy resolution to data by one standard deviation around its central value, resulting in an uncertainty of up to 1–2%.
- Efficiency correction:
  The uncertainty coming from the measurements of trigger efficiency, lepton reconstruction, and lepton identification is estimated by varying the scale factors by their uncertainties, as described in Ref. [5]. The resulting uncertainty in the differential cross section measurement is less than 1%.
- Luminosity:
  The uncertainty in the integrated luminosity is 1.2% [68]. It is applied as a global scale factor to the cross section as well as to the normalization of the background samples.
- Pileup:
  The determination of the simulated pileup profile is based on a total inelastic pp cross section of 69.2 mb [69]. Alternative pileup profiles are generated by varying this cross section by 5% affecting the measurement by 1–2%.
- Prefiring:
  The prefiring uncertainty is estimated by up and down variations of the prefiring weight. The uncertainty is less than 0.5%.
- Background:
  The theoretical uncertainty in the calculation of background processes is used to estimate the uncertainty in the background modeling. The main source of background is $\text {t}\overline{\text {t}}$ production on this process. An uncertainty of around 6% is estimated using the TOP++2.0 program, which includes scale and PDF variations. The resulting uncertainty is less than 0.2%. Systematic uncertainties stemming from other background processes are negligible.
- Unfolding and model:
  The uncertainty of unfolding and modeling is estimated by reweighting the simulated signal event sample to match the data and using this as an alternative model for unfolding. This gives an uncertainty of about 2%. The uncertainty coming from the finite size of the simulation sample that is used to correct the data for detector effects results in an uncertainty of 2–8%.

A summary table of these uncertainties is given in Table 3. All the systematic uncertainties are quadratically summed assuming independent uncertainty sources.

**Table 2:** Particle-level phase space definition

Object	requirement
Leading (subleading) lepton	$p_{\textrm{T}} >25(20)\,\text {Ge}\hspace{-.08em}\text {V}$, $|\eta |<2.4$
Lepton-jet separation	$\varDelta R_{\ell ,j}>0.4$
Lepton pair mass	$76<m_{\ell ^+ \ell ^-}<106\,\text {Ge}\hspace{-.08em}\text {V}$
Jet	$p_{\textrm{T}} >30\,\text {Ge}\hspace{-.08em}\text {V}$, $|\eta _{jet} |<2.4$

**Table 3:** Systematic uncertainties on the unfolded differential cross section

Uncertainty source	(%)
Jet energy scale	3–7
Jet energy resolution	1–2
Efficiency correction	<1
Luminosity	1.2
Pileup	1–2
Prefiring	$<0.5$
Background	$<0.2$
Unfolding and model	2–8
Total	4–11

## Results

The production cross section of Z +jets is measured in the phase space given in Table 2. The Z boson is identified via its leptonic decay channel. The results of the muon and electron decay channels are combined using the best linear unbiased estimator (BLUE) [70, 71] approach.

**Fig. 2:**
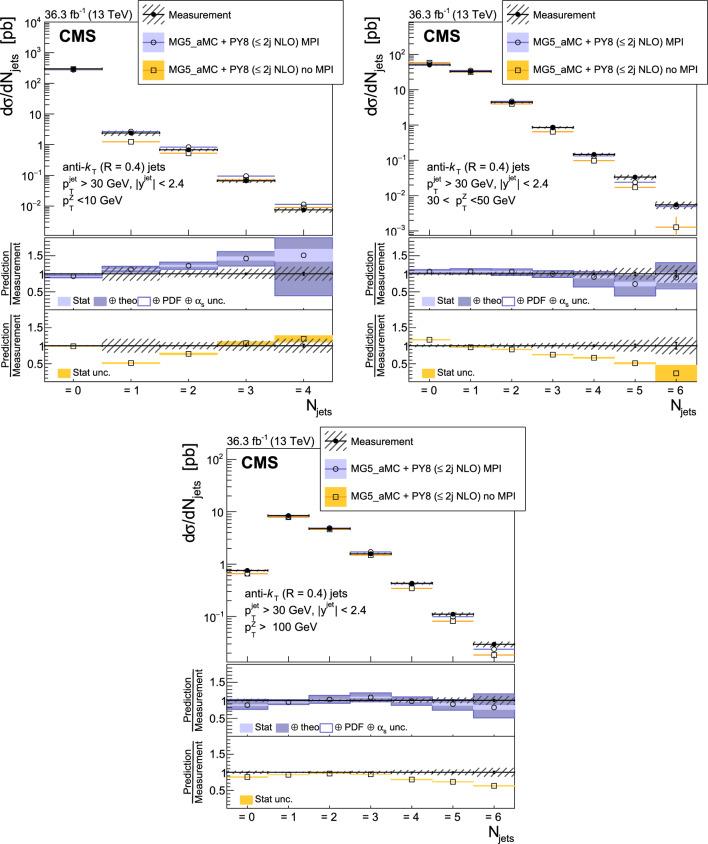
Jet multiplicity in three different regions of $p_{\textrm{T}} (\text {Z })$: $p_{\textrm{T}} (\text {Z }) <10\,\text {Ge}\hspace{-.08em}\text {V}$ (upper left), $30< p_{\textrm{T}} (\text {Z }) < 50\,\text {Ge}\hspace{-.08em}\text {V}$ (upper right), $p_{\textrm{T}} (\text {Z }) > 100\,\text {Ge}\hspace{-.08em}\text {V}$ (lower). The error bars on the data points represent the statistical uncertainty of the measurement, and the hatched band shows the total statistical and systematic uncertainties added in quadrature. Predictions using MG5_aMC+Py8 ($\le 2j$ NLO) with and without MPI are shown

In Fig. 2, the exclusive jet multiplicity is shown for three different ranges of $p_{\textrm{T}} (\text {Z })$. At low $p_{\textrm{T}} (\text {Z })$, the majority of events have no jet with $p_{\textrm{T}} > 30\,\text {Ge}\hspace{-.08em}\text {V}$ and only about 1% of the events have one or more jets. This suggests that the $p_{\textrm{T}} (\text {Z })$ is mainly compensated by softer ($p_{\textrm{T}} < 30\,\text {Ge}\hspace{-.08em}\text {V}$) radiation at low $p_{\textrm{T}} (\text {Z })$. Events with higher jet multiplicity indicate that the dominant hard process is essentially a jet production process, and the Z boson is radiated from a quark, as an electroweak correction to a pure QCD process. At high $p_{\textrm{T}} (\text {Z })$, the majority of events have at least one jet with a tail towards higher jet multiplicities, which indicates that the hardest process is indeed Z +jet, and additional jets originate from higher-order QCD corrections.

In Fig. 2, the measurement is compared with the generator MG5_aMC+Py8 ($\le 2j$ NLO) with and without multiparton interactions. The MPI contribution is important in the low $p_{\textrm{T}} (\text {Z })$ region, but also at higher $p_{\textrm{T}} (\text {Z })$ and higher jet multiplicities MPI plays a role. The prediction of MG5_aMC+Py8 ($\le 2j$ NLO) including MPI agrees with the measurement, even for high jet multiplicities. This behaviour is consistent with the prediction of the dependence of MPI effects on event kinematics from the $p_{\textrm{T}} (\text {Z })$ reported in [72].

**Fig. 3:**
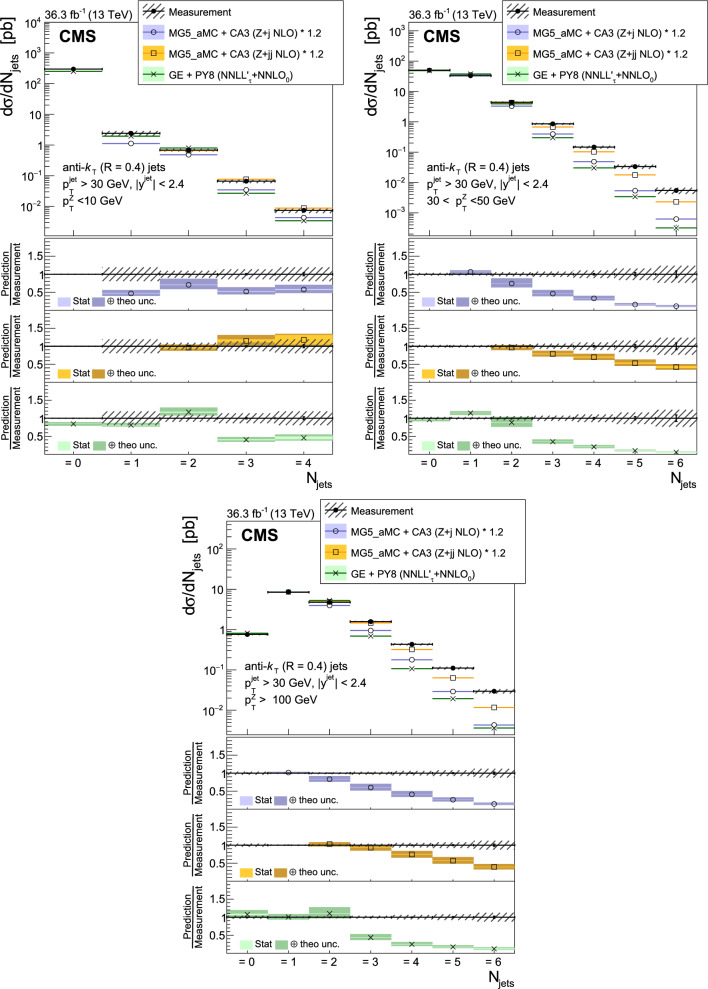
Jet multiplicity in three different regions of $p_{\textrm{T}} (\text {Z })$: $p_{\textrm{T}} (\text {Z }) <10\,\text {Ge}\hspace{-.08em}\text {V}$ (upper left), $30< p_{\textrm{T}} (\text {Z }) < 50\,\text {Ge}\hspace{-.08em}\text {V}$ (upper right), $p_{\textrm{T}} (\text {Z }) > 100\,\text {Ge}\hspace{-.08em}\text {V}$ (lower). The error bars on the data points represent the statistical uncertainty of the measurement, and the hatched band shows the total statistical and systematic uncertainties added in quadrature. Predictions from MG5_aMC+CA3 (Z +1 NLO), MG5_aMC+CA3 (Z +2 NLO) and Geneva (Z +0 NNLO) are shown. An overall normalization factor of 1.2 is applied to MG5_aMC+CA3 (Z +1 NLO) and MG5_aMC+CA3 (Z +2 NLO)

In Fig. 3, a comparison of the measurement with predictions from MG5_aMC+CA3 (Z +1 NLO), MG5_aMC+CA3 (Z +2 NLO) and Geneva (Z +0 NNLO) is shown. Both MG5_aMC+CA3 (Z +1 NLO) and MG5_aMC+CA3 (Z +2 NLO) predictions are multiplied by a factor 1.2 to account for the normalization of PB TMD set 2 (as discussed in Sect. 3). For $p_{\textrm{T}} (\text {Z }) > 30\,\text {Ge}\hspace{-.08em}\text {V}$ the Z +1 (Z +2) predictions describe well the one (two) jet multiplicities, whereas at higher multiplicities a deviation from these measurement is observed, which can be attributed to the missing MPI contributions (as shown in Fig. 2). The Geneva (Z +0 NNLO) predictions, which include MPI, are in agreement for low jet multiplicities for low $p_{\textrm{T}} (\text {Z })$, whereas higher jet multiplicities are not well described because of missing higher order contributions in the ME calculations.

**Fig. 4:**
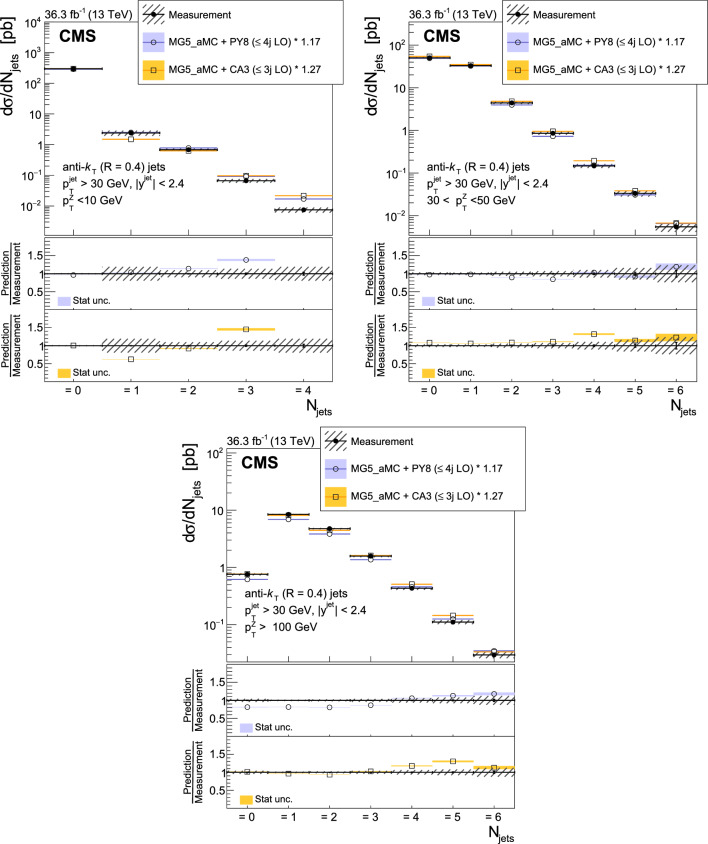
Jet multiplicity in three different regions of $p_{\textrm{T}} (\text {Z })$: $p_{\textrm{T}} (\text {Z }) <10\,\text {Ge}\hspace{-.08em}\text {V}$ (upper left), $30< p_{\textrm{T}} (\text {Z }) < 50\,\text {Ge}\hspace{-.08em}\text {V}$ (upper right), $p_{\textrm{T}} (\text {Z }) > 100\,\text {Ge}\hspace{-.08em}\text {V}$ (lower). The error bars on the data points represent the statistical uncertainty of the measurement, and the hatched band shows the total statistical and systematic uncertainties added in quadrature. Predictions from MG5_aMC+Py8 ($\le 4j$ LO) and MG5_aMC+CA3 (Z $\le 3j$ LO) are shown. Different normalization factors are applied, as described in the text

In Fig. 4, the measurement is compared with predictions from MG5_aMC+Py8 ($\le 4j$ LO) and MG5_aMC+CA3 (Z $\le 3j$ LO). The prediction from MG5_aMC+Py8 ($\le 4j$ LO) describes the measurements in all $p_{\textrm{T}} (\text {Z })$ regions. The MG5_aMC+CA3 (Z $\le 3j$ LO) prediction agrees with the measurements in all $p_{\textrm{T}} (\text {Z })$ ranges, except in the second bin at low $p_{\textrm{T}} (\text {Z })$ values where MPI plays a significant role.

**Fig. 5:**
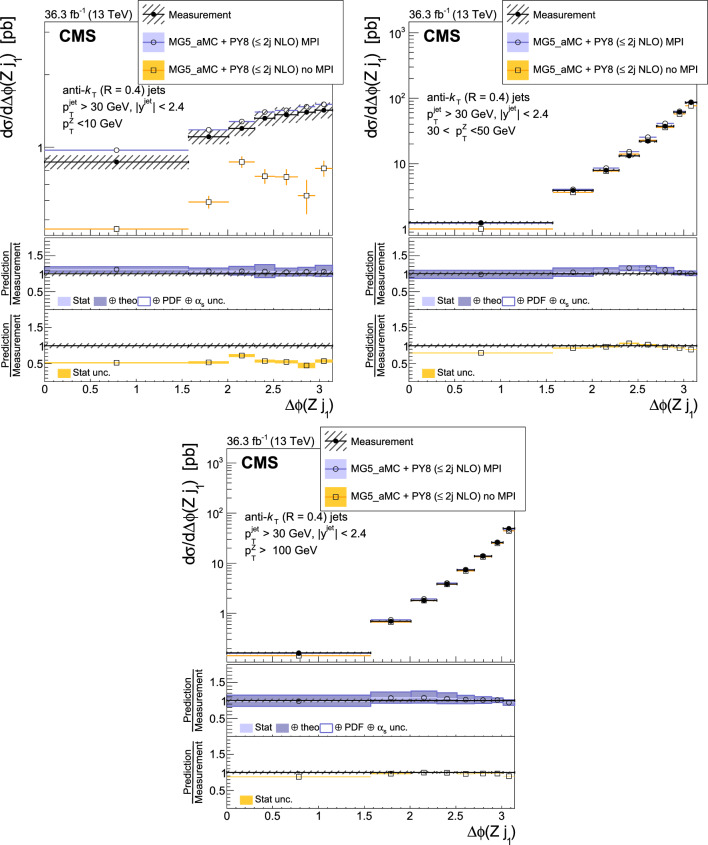
Cross section as a function of $\varDelta \phi (\text {Z }j_1)$ between the Z boson and the leading jet in the three $p_{\textrm{T}} (\text {Z })$ bins: $p_{\textrm{T}} (\text {Z }) <10\,\text {Ge}\hspace{-.08em}\text {V}$ (upper left), $30< p_{\textrm{T}} (\text {Z }) < 50\,\text {Ge}\hspace{-.08em}\text {V}$ (upper right), $p_{\textrm{T}} (\text {Z }) > 100\,\text {Ge}\hspace{-.08em}\text {V}$ (lower). The error bars on the data points represent the statistical uncertainty of the measurement, and the hatched band shows the total statistical and systematic uncertainties added in quadrature. Predictions using MG5_aMC+Py8 ($\le 2j$ NLO) with and without multi-parton interactions are shown

In Fig. 5, the azimuthal correlation, $\varDelta \phi (\text {Z }j_1)$, between the Z boson and the leading jet is shown for three different ranges of $p_{\textrm{T}} (\text {Z })$. In the range $p_{\textrm{T}} (\text {Z }) < 10\,\text {Ge}\hspace{-.08em}\text {V}$, the Z boson is only very weakly correlated with the leading jet, thus the distribution is almost uniform. In the region $p_{\textrm{T}} (\text {Z }) > 100\,\text {Ge}\hspace{-.08em}\text {V}$, the Z boson is highly correlated with the leading jet and the cross section falls more than two orders of magnitude from the back-to-back region to the small $\varDelta \phi (\text {Z }j_1)$ region. The systematic uncertainty in the low $p_{\textrm{T}} (\text {Z })$ range is $\mathcal {O}(10 \%)$. In Fig. 5, the predictions from MG5_aMC+Py8 ($\le 2j$ NLO) with and without MPI are compared with the measurement. In the low $p_{\textrm{T}} (\text {Z })$ range, MPI contributes about 40%, and even in the region of $30< p_{\textrm{T}} (\text {Z }) < 50\,\text {Ge}\hspace{-.08em}\text {V}$, the contribution from MPI could be about 20% in the small-$\varDelta \phi (\text {Z }j_1)$ region. The prediction of MG5_aMC+Py8 ($\le 2j$ NLO) including MPI describes the measurements.

**Fig. 6:**
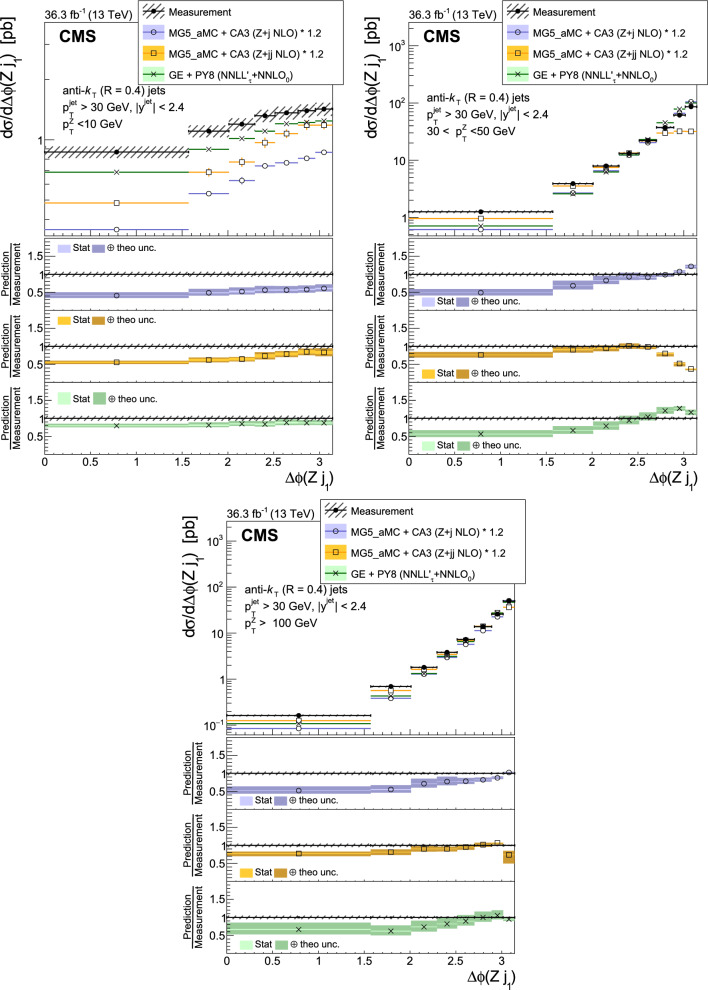
Cross section as a function of $\varDelta \phi (\text {Z }j_1)$ between the Z boson and the leading jet in the three $p_{\textrm{T}} (\text {Z })$ bins: $p_{\textrm{T}} (\text {Z }) <10\,\text {Ge}\hspace{-.08em}\text {V}$ (upper left), $30< p_{\textrm{T}} (\text {Z }) < 50\,\text {Ge}\hspace{-.08em}\text {V}$ (upper right), $p_{\textrm{T}} (\text {Z }) > 100\,\text {Ge}\hspace{-.08em}\text {V}$ (lower). The error bars on the data points represent the statistical uncertainty of the measurement, and the hatched band shows the total statistical and systematic uncertainties added in quadrature. Predictions from Geneva (Z +0 NNLO), MG5_aMC+CA3 (Z +1 NLO) and MG5_aMC+CA3 (Z +2 NLO) are shown. An overall normalization factor of 1.2 is applied to MG5_aMC+CA3 (Z +1 NLO) and MG5_aMC+CA3 (Z +2 NLO)

In Fig. 6, the measurement is compared with MG5_aMC+CA3 (Z +1 NLO), MG5_aMC+CA3 (Z +2 NLO) and Geneva (Z +0 NNLO). In low $p_{\textrm{T}} (\text {Z })$ range, the MG5_aMC+CA3 (Z +1 NLO) and MG5_aMC+CA3 (Z +2 NLO) predictions differ from the measurements due to the missing contribution of MPI. In the high $p_{\textrm{T}} (\text {Z })$ region the predictions agree better with the measurements (the region $\varDelta \phi (\text {Z }j_1) \rightarrow \pi$ is not accessible in the Z +2 calculation). The Geneva (Z +0 NNLO) prediction agrees with the measurement at low $p_{\textrm{T}} (\text {Z })$, whereas at larger $p_{\textrm{T}} (\text {Z })$ the prediction differs from the measurement because of missing higher order contributions.

**Fig. 7:**
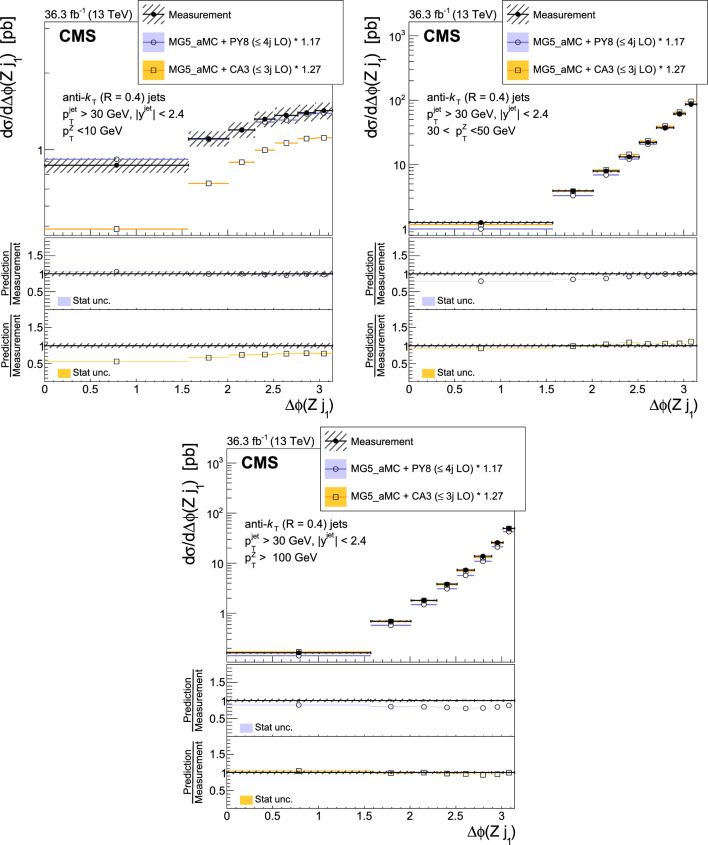
Cross section as a function of $\varDelta \phi (\text {Z }j_1)$ between the Z boson and the leading jet in three $p_{\textrm{T}} (\text {Z })$ bins: $p_{\textrm{T}} (\text {Z }) <10\,\text {Ge}\hspace{-.08em}\text {V}$ (upper left), $30< p_{\textrm{T}} (\text {Z }) < 50\,\text {Ge}\hspace{-.08em}\text {V}$ (upper right), $p_{\textrm{T}} (\text {Z }) > 100\,\text {Ge}\hspace{-.08em}\text {V}$ (lower). The error bars on the data points represent the statistical uncertainty of the measurement, and the hatched band shows the total statistical and systematic uncertainties added in quadrature. Predictions from MG5_aMC+Py8 ($\le 4j$ LO) and MG5_aMC+CA3 (Z $\le 3j$ LO) are shown. Different normalization factors are applied, as described in the text

In Fig. 7, the predictions from MG5_aMC+Py8 ($\le 4j$ LO) and MG5_aMC+CA3 (Z $\le 3j$ LO) are compared with the measurement. The MG5_aMC+Py8 ($\le 4j$ LO) is in agreement with the measurement. The MG5_aMC+CA3 (Z $\le 3j$ LO) prediction is too low in the low $p_{\textrm{T}} (\text {Z })$ region, due to the missing MPI contribution, whereas other $p_{\textrm{T}} (\text {Z })$ ranges are described.

**Fig. 8:**
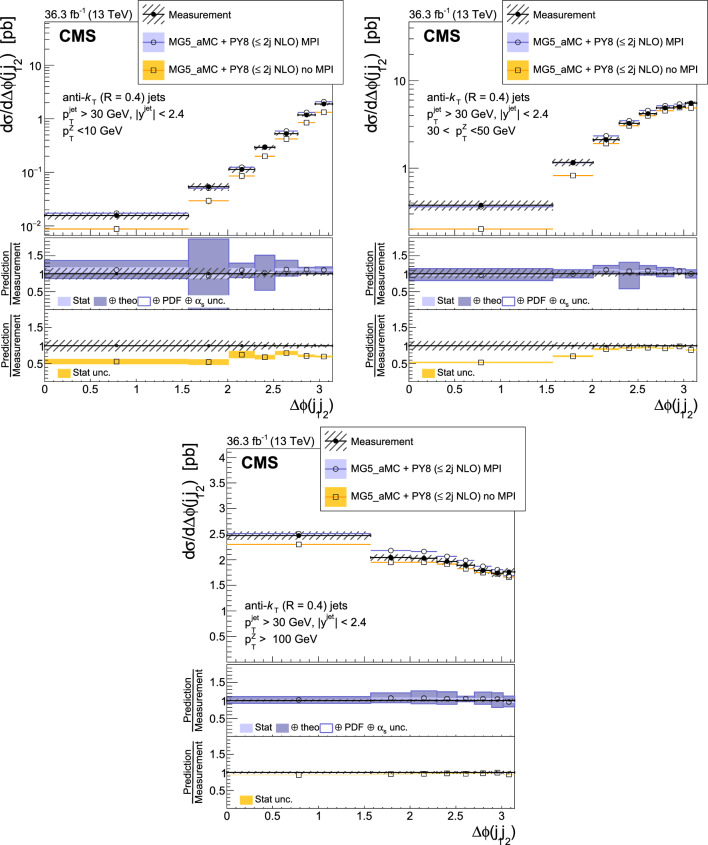
Cross section as a function of $\varDelta \phi (j_1j_2)$ between two leading jets in three $p_{\textrm{T}} (\text {Z })$ regions: $p_{\textrm{T}} (\text {Z }) <10\,\text {Ge}\hspace{-.08em}\text {V}$ (upper left), $30< p_{\textrm{T}} (\text {Z }) < 50\,\text {Ge}\hspace{-.08em}\text {V}$ (upper right), $p_{\textrm{T}} (\text {Z }) > 100\,\text {Ge}\hspace{-.08em}\text {V}$ (lower). The error bars on the data points represent the statistical uncertainty of the measurement, and the hatched band shows the total statistical and systematic uncertainties added in quadrature. Predictions using MG5_aMC+Py8 ($\le 2j$ NLO) with and without multiparton interactions are shown

In Fig. 8, the azimuthal correlation $\varDelta \phi (j_1j_2)$ between the two leading jets is shown for the three different ranges of $p_{\textrm{T}} (\text {Z })$. A strong correlation between the two leading jets is observed at small $p_{\textrm{T}} (\text {Z })$, whereas only a weak correlation is seen at large $p_{\textrm{T}} (\text {Z })$. This indicates that at low $p_{\textrm{T}} (\text {Z })$ the process is dominated by a jet production process and that the Z boson is radiated from a quark (EW correction) and therefore the jets are correlated. On the contrary, at large $p_{\textrm{T}} (\text {Z })$ the process is dominated by Z +jet production, with higher-order QCD corrections in form of additional jets, which are only weakly correlated. The measurement is compared with MG5_aMC+Py8 ($\le 2j$ NLO), with and without MPI. Except for the highest $p_{\textrm{T}} (\text {Z })$ region, the contribution from MPI is significant, especially in the small $\varDelta \phi (j_1j_2)$ range. The prediction obtained with MG5_aMC+Py8 ($\le 2j$ NLO) including MPI describes the measurement well over the whole range.

**Fig. 9:**
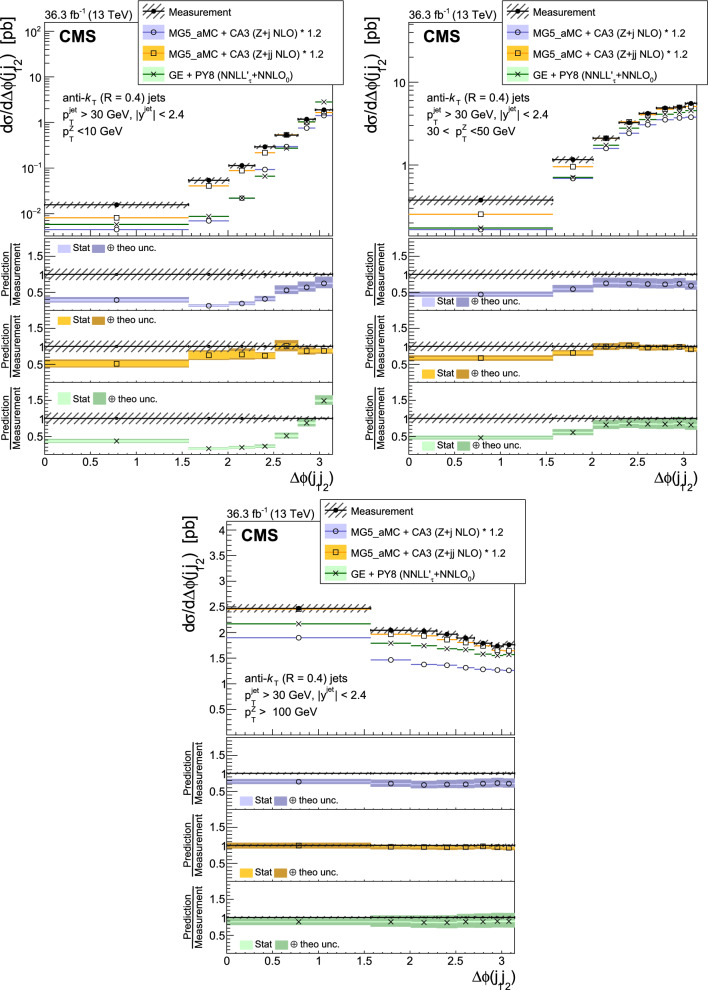
Cross section as a function of $\varDelta \phi (j_1j_2)$ between two leading jets in three $p_{\textrm{T}} (\text {Z })$ regions: $p_{\textrm{T}} (\text {Z }) <10\,\text {Ge}\hspace{-.08em}\text {V}$ (upper left), $30< p_{\textrm{T}} (\text {Z }) < 50\,\text {Ge}\hspace{-.08em}\text {V}$ (upper right), $p_{\textrm{T}} (\text {Z }) > 100\,\text {Ge}\hspace{-.08em}\text {V}$ (lower). The error bars on the data points represent the statistical uncertainty of the measurement, and the hatched band shows the total statistical and systematic uncertainties added in quadrature. Predictions from MG5_aMC+CA3 (Z +1 NLO), MG5_aMC+CA3 (Z +2 NLO) and Geneva (Z +0 NNLO) are shown. An overall normalization factor of 1.2 is applied to the MG5_aMC+CA3 (Z +1 NLO) and MG5_aMC+CA3 (Z +2 NLO) predictions

**Fig. 10:**
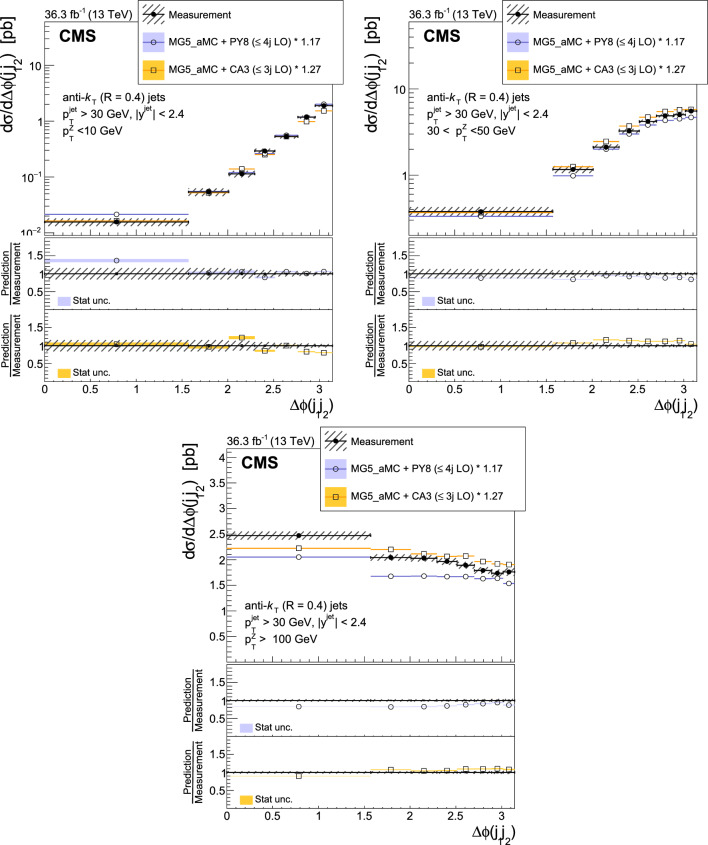
Cross section as a function of $\varDelta \phi (j_1j_2)$ between two leading jets in three $p_{\textrm{T}} (\text {Z })$ regions: $p_{\textrm{T}} (\text {Z }) <10\,\text {Ge}\hspace{-.08em}\text {V}$ (upper left), $30< p_{\textrm{T}} (\text {Z }) < 50\,\text {Ge}\hspace{-.08em}\text {V}$ (upper right), $p_{\textrm{T}} (\text {Z }) > 100\,\text {Ge}\hspace{-.08em}\text {V}$ (lower). The error bars on the data points represent the statistical uncertainty of the measurement, and the hatched band shows the total statistical and systematic uncertainties added in quadrature. Predictions from MG5_aMC+Py8 ($\le 4j$ LO) and MG5_aMC+CA3 (Z $\le 3j$ LO) are shown. Different normalization factors are applied, as described in the text

In Fig. 9, the predictions from MG5_aMC+CA3 (Z +1 NLO), MG5_aMC+CA3 (Z +2 NLO), and Geneva (Z +0 NNLO) are shown. In general, the MG5_aMC+CA3 (Z +1 NLO) prediction is not sufficient to describe the measurement, whereas the MG5_aMC+CA3 (Z +2 NLO) prediction describes the measurements at high $p_{\textrm{T}} (\text {Z })$, where MPI effects are negligible. At lower $p_{\textrm{T}} (\text {Z })$, MPI effects become important, as shown in Fig. 8. The Geneva (Z +0 NNLO) prediction is below the measurement at low $p_{\textrm{T}} (\text {Z })$ because of missing higher order contributions, as is the prediction from MG5_aMC+CA3 (Z +1 NLO).

In Fig. 10, the predictions from MG5_aMC+Py8 ($\le 4j$ LO) and MG5_aMC+CA3 (Z $\le 3j$ LO) are compared with the measurement. The MG5_aMC+Py8 ($\le 4j$ LO) prediction agrees with the measurement. The MG5_aMC+CA3 (Z $\le 3j$ LO) prediction describes the measurement in certain $p_{\textrm{T}} (\text {Z })$ regions.

The contribution from MPI is significant in the low $p_{\textrm{T}} (\text {Z })$ regions and becomes negligible when $p_{\textrm{T}} (\text {Z }) > 100\,\text {Ge}\hspace{-.08em}\text {V}$. The calculation MG5_aMC+Py8 ($\le 2j$ NLO) describes the measurements within the scale uncertainties, if an appropriate tune for PS and underlying event parameters are applied (here, the CUETP8M1 tune).

The predictions of MG5_aMC+CA3 using PB-TMD PDFs and initial-state PB PS come remarkably close to the measurements in regions of phase space where they are applicable. The prediction of Geneva (Z +0 NNLO) for Z +jet observables describe the measurements in the regions of low jet multiplicity but show differences when two or more jets are selected since higher order contributions are not included. The prediction of the merged LO calculations MG5_aMC+Py8 ($\le 4j$ LO) and MG5_aMC+CA3 (Z $\le 3j$ LO) describe the measurements quite well keeping in mind the role of MPI at low $p_{\textrm{T}} (\text {Z })$.

In Ref. [73], calculations using TMDs and the “winner-takes-all” jet recombination scheme for the azimuthal angular decorrelation in Z +jet are described. The calculations reported here do not change significantly if the “winner-takes-all” recombination scheme in the anti-$k_{\textrm{T}}$ algorithm is applied.

In this paper the differential cross section measurements are presented in three representative regions of the Z boson transverse momentum, the results for intervals $10<p_{\textrm{T}} (\text {Z }) <30\,\text {Ge}\hspace{-.08em}\text {V}$ and $50<p_{\textrm{T}} (\text {Z }) <100\,\text {Ge}\hspace{-.08em}\text {V}$ can be also found in HEPData [74].

## Summary

We have measured the Z +jet production cross section in proton–proton collisions at the LHC at a center-of-mass energy of 13$\,\text {Te}\hspace{-.08em}\text {V}$. The associated jet multiplicity for various regions of the transverse momentum of the Z boson, $p_{\textrm{T}} (\text {Z })$, was measured. At $p_{\textrm{T}} (\text {Z }) < 10\,\text {Ge}\hspace{-.08em}\text {V}$ only about 1% of the events have jets with $p_{\textrm{T}} > 30\,\text {Ge}\hspace{-.08em}\text {V}$, with nonnegligible cross sections at high jet multiplicity. At $30< p_{\textrm{T}} (\text {Z }) < 50\,\text {Ge}\hspace{-.08em}\text {V}$, most of the events have at least one jet, with a significant tail to higher jet multiplicities. The azimuthal angle $\varDelta \phi (\text {Z }j_1)$ between the Z boson and the leading jet, as well as the azimuthal angle $\varDelta \phi (j_1j_2)$ between the two leading jets, was measured for the three $p_{\textrm{T}} (\text {Z })$ regions. At low $p_{\textrm{T}} (\text {Z })$, the Z boson is only loosely correlated with the jets, but the two leading jets are strongly correlated. At large $p_{\textrm{T}} (\text {Z })$, the Z boson is highly correlated with the leading jet, but the two leading jets are only weakly correlated.

The measurement shows that at low $p_{\textrm{T}} (\text {Z })$ the Z boson appears as an electroweak correction to high-$p_{\textrm{T}}$ jet production, whereas at large $p_{\textrm{T}} (\text {Z })$ the dominant process is Z +jet production.

The next-to-leading order (NLO) prediction of MG5_aMC+Py8 ($\le 2j$ NLO) with Z +0,1,2 partons, which is merged with the FxFx procedure and supplemented with parton showering (PS) and multiple parton interactions (MPI) from pythia 8, agrees with the measurements.

The predictions of MG5_aMC+CA3 (Z +1 NLO) and MG5_aMC+CA3 (Z +2 NLO) using the parton branching method with transverse-momentum dependent (PB-TMD) parton densities, which do not include MPI effects, and the corresponding PS agree with the measurements in the regions where MPI effects are negligible. The prediction from Geneva (Z +0 NNLO) using matrix elements at next-to-next-to-leading order for Z production, supplemented with resummation, PS and MPI from pythia 8, agrees with the measurements in the low jet multiplicity region.

The leading order prediction of MG5_aMC+Py8 ($\le 4j$ LO), including merging of jet multiplicities, describes the measurements well. The prediction of MG5_aMC+CA3 (Z $\le 3j$ LO) using PB-TMD parton densities and PS with merging of jet multiplicities agrees well with the measurements in the regions where MPI is negligible.

In summary, Z +jet measurements challenge theoretical predictions; a good agreement can be achieved by including contributions of multiparton interactions, parton showering, parton densities, as well as multijet matrix element merging. The differential measurements provided here help to disentangle the various contributions and illustrate where each contribution becomes important.

## Data Availability

This manuscript has no associated data or the data will not be deposited. [Authors’ comment: Release and preservation of data used by the CMS Collaboration as the basis for publications is guided by the CMS policy as stated in https://cms-docdb.cern.ch/cgi-bin/PublicDocDB/RetrieveFile?docid=6032 &filename=CMSDataPolicyV1.2.pdf &version=2. CMS data preservation, re-use and open access policy.]

## References

[1] Ellis RK, Martinelli G, Petronzio R (1983). Lepton pair production at large transverse momentum in second order QCD. Nucl. Phys. B.

[2] Collins JC, Soper DE, Sterman G (1985). Transverse momentum distribution in Drell–Yan pair and W and Z boson production. Nucl. Phys. B.

[3] Bizon W (2019). The transverse momentum spectrum of weak gauge bosons at N $${}^3$$ LL + NNLO. Eur. Phys. J. C.

[4] Bermudez Martinez A (2019). Production of Z-bosons in the parton branching method. Phys. Rev. D.

[5] Collaboration CMS (2019). Measurements of differential Z boson production cross sections in proton-proton collisions at $$ \sqrt{s} = 13$$ TeV. JHEP.

[6] CDF Collaboration, Measurement of inclusive jet cross-sections in $${\text{Z}}/\gamma ^* \rightarrow {{\text{ e}^{+}\text{ e}^{-}}}$$ + jets production in $${\rm p}\bar{{\rm p}}$$ collisions at $$\sqrt{s}$$ = 1.96 TeV. Phys. Rev. Lett. **100**, 102001 (2008). 10.1103/PhysRevLett.100.102001. arXiv:0711.3717 10.1103/PhysRevLett.100.102001 18352174

[7] D0 Collaboration, Measurement of differential $${\text{ Z }}/\gamma ^{*}$$ + jet + $$X$$ cross sections in $${\rm p}\bar{{\rm p}}$$ collisions at $$\sqrt{s}$$ = 1.96 TeV. Phys. Lett. B **669**, 278 (2008). 10.1016/j.physletb.2008.09.060. arXiv:0808.1296

[8] ATLAS Collaboration, Measurement of the production cross section of jets in association with a Z boson in pp collisions at $$\sqrt{s} =$$ 7 TeV with the ATLAS detector. JHEP **07**, 032 (2013). 10.1007/JHEP07(2013)032. arXiv:1304.7098

[9] ATLAS Collaboration, Measurement of the production cross section for $${\text{ Z }}/\gamma ^*$$ in association with jets in $${\rm pp}$$ collisions at $$\sqrt{s}=7$$ TeV with the ATLAS detector. Phys. Rev. D **85**, 032009 (2012). 10.1103/PhysRevD.85.032009. arXiv:1111.2690

[10] Collaboration CMS (2015). Measurements of jet multiplicity and differential production cross sections of $$Z+$$jets events in proton–proton collisions at $$\sqrt{s} =$$ 7 TeV. Phys. Rev. D.

[11] Collaboration CMS (2012). Jet production rates in association with $${\rm W}$$ and $${\rm Z}$$ bosons in $${\rm pp}$$ collisions at $$\sqrt{s}=7$$ TeV. JHEP.

[12] Collaboration CMS (2013). Event shapes and azimuthal correlations in $${\rm Z}$$+jets events in $${\rm pp}$$ collisions at $$\sqrt{s}=7$$ TeV. Phys. Lett. B.

[13] LHCb Collaboration, Study of forward Z+jet production in pp collisions at $$\sqrt{s} = 7$$ TeV. JHEP **01**, 033 (2014). 10.1007/JHEP01(2014)033. arXiv:1310.8197

[14] Collaboration CMS (2017). Measurements of differential production cross sections for a Z boson in association with jets in pp collisions at $$ \sqrt{s}=$$ 8 TeV. JHEP.

[15] ATLAS Collaboration, Measurement of the inclusive cross-section for the production of jets in association with a Z boson in proton–proton collisions at 8 TeV using the ATLAS detector. Eur. Phys. J. C **79**, 847 (2019). 10.1140/epjc/s10052-019-7321-3. arXiv:1907.06728

[16] CMS Collaboration, Measurement of differential cross sections for Z boson production in association with jets in proton–proton collisions at $$\sqrt{s} =$$ 13 TeV. Eur. Phys. J. C **78**, 965 (2018). 10.1140/epjc/s10052-018-6373-0. arXiv:1804.05252 10.1140/epjc/s10052-018-6373-0 PMC6394299 30881214

[17] ATLAS Collaboration, Measurements of the production cross section of a $$Z$$ boson in association with jets in pp collisions at $$\sqrt{s} = 13$$ TeV with the ATLAS detector. Eur. Phys. J. C **77**, 361 (2017). 10.1140/epjc/s10052-017-4900-z. arXiv:1702.05725 10.1140/epjc/s10052-017-4900-z PMC5689544 29200941

[18] Hautmann F (2018). Collinear and TMD quark and gluon densities from parton branching solution of QCD evolution equations. JHEP.

[19] Hautmann F (2017). Soft-gluon resolution scale in QCD evolution equations. Phys. Lett. B.

[20] A. Bermudez Martinez et al., Collinear and TMD parton densities from fits to precision DIS measurements in the parton branching method. Phys. Rev. D **99**, 074008 (2019). 10.1103/PhysRevD.99.074008. arXiv:1804.11152

[21] Baranov S (2021). CASCADE3 A Monte Carlo event generator based on TMDs. Eur. Phys. J. C.

[22] Alioli S, Bauer CW, Guns S, Tackmann FJ (2016). Underlying event sensitive observables in Drell–Yan production using GENEVA. Eur. Phys. J. C.

[23] Alioli S (2015). Drell–Yan production at NNLL$$^{\prime }$$+NNLO matched to parton showers. Phys. Rev. D.

[24] Alioli S (2014). Matching fully differential NNLO calculations and parton showers. JHEP.

[25] Alioli S (2013). Combining higher-order resummation with multiple NLO calculations and parton showers in GENEVA. JHEP.

[26] CMS Collaboration, Performance of the CMS Level-1 trigger in proton–proton collisions at $$\sqrt{s} = 13$$ TeV. JINST **15**, P10017 (2020). 10.1088/1748-0221/15/10/P10017. arXiv:2006.10165

[27] CMS Collaboration, The CMS trigger system. JINST **12**, P01020 (2017). 10.1088/1748-0221/12/01/P01020. arXiv:1609.02366

[28] CMS Collaboration, Particle-flow reconstruction and global event description with the CMS detector. JINST **12**, P10003 (2017). 10.1088/1748-0221/12/10/P10003. arXiv:1706.04965

[29] CMS Collaboration, Technical proposal for the Phase-II upgrade of the Compact Muon Solenoid. CMS Technical Proposal CERN-LHCC-2015-010, CMS-TDR-15-02 (2015). 10.17181/CERN.VU8I.D59J

[30] CMS Collaboration, ECAL 2016 refined calibration and Run2 summary plots. CMS Detector Performance Summary CMS-DP-2020-021 (2020). https://cds.cern.ch/record/2717925

[31] CMS Collaboration, Electron and photon reconstruction and identification with the CMS experiment at the CERN LHC. JINST **16**, P05014 (2021). 10.1088/1748-0221/16/05/p05014. arXiv:2012.06888

[32] CMS Collaboration, Performance of the CMS muon detector and muon reconstruction with proton-proton collisions at $$\sqrt{s}=$$ 13 TeV. JINST **13**, P06015 (2018). 10.1088/1748-0221/13/06/P06015. arXiv:1804.04528

[33] Cacciari M, Salam GP, Soyez G (2008). The anti-$$k_{\rm T }$$ jet clustering algorithm. JHEP.

[34] Cacciari M, Salam GP, Soyez G (2012). FastJet user manual. Eur. Phys. J. C.

[35] CMS Collaboration, Jet energy scale and resolution in the CMS experiment in pp collisions at 8 TeV. JINST **12**, P02014 (2017). 10.1088/1748-0221/12/02/P02014. arXiv:1607.03663

[36] CMS Collaboration, The CMS experiment at the CERN LHC. JINST **3**, S08004 (2008). 10.1088/1748-0221/3/08/S08004

[37] Alwall J (2014). The automated computation of tree-level and next-to-leading order differential cross sections, and their matching to parton shower simulations. JHEP.

[38] Sjöstrand T (2015). An introduction to PYTHIA 82. Comput. Phys. Commun..

[39] Dokshitzer YL, Khoze VA, Troian SI, Mueller AH (1988). QCD coherence in high-energy reactions. Rev. Mod. Phys..

[40] Bassetto A, Ciafaloni M, Marchesini G (1983). Jet structure and infrared sensitive quantities in perturbative QCD. Phys. Rep..

[41] Marchesini G, Webber BR (1988). Monte Carlo simulation of general hard processes with coherent QCD radiation. Nucl. Phys. B.

[42] Catani S, Webber BR, Marchesini G (1991). QCD coherent branching and semiinclusive processes at large $$x$$. Nucl. Phys. B.

[43] NNPDF Collaboration, Parton distributions for the LHC Run II. JHEP **04**, 040 (2015). 10.1007/JHEP04(2015)040. arXiv:1410.8849

[44] CMS Collaboration, Event generator tunes obtained from underlying event and multiparton scattering measurements. Eur. Phys. J. C **76**, 155 (2016). 10.1140/epjc/s10052-016-3988-x. arXiv:1512.00815 10.1140/epjc/s10052-016-3988-x PMC4946872 27471433

[45] Frederix R, Frixione S (2012). Merging meets matching in MC@NLO. JHEP.

[46] Alwall J (2008). Comparative study of various algorithms for the merging of parton showers and matrix elements in hadronic collisions. Eur. Phys. J. C.

[47] NNPDF Collaboration, Parton distributions with QED corrections. Nucl. Phys. B **877**, 290 (2013). 10.1016/j.nuclphysb.2013.10.010. arXiv:1308.0598

[48] NNPDF Collaboration, Unbiased global determination of parton distributions and their uncertainties at NNLO and at LO. Nucl. Phys. B **855**, 153 (2012). 10.1016/j.nuclphysb.2011.09.024. arXiv:1107.2652

[49] Li Y, Petriello F (2012). Combining QCD and electroweak corrections to dilepton production in the framework of the FEWZ simulation code. Phys. Rev. D.

[50] T. Sjöstrand, S. Mrenna, P. Skands, PYTHIA 6.4 physics and manual. JHEP **05**, 026 (2006). 10.1088/1126-6708/2006/05/026. arXiv:hep-ph/0603175

[51] A. Bermudez Martinez, F. Hautmann, M.L. Mangano, TMD evolution and multi-jet merging. Phys. Lett. B **822**, 136700 (2021). 10.1016/j.physletb.2021.136700. arXiv:2107.01224

[52] Butterworth J (2016). PDF4lhc recommendations for LHC run II. J. Phys. G Nucl. Part. Phys..

[53] Alwall J (2011). MadGraph 5: going beyond. JHEP.

[54] NNPDF Collaboration, Parton distributions from high-precision collider data. Eur. Phys. J. C **77**, 663 (2017). 10.1140/epjc/s10052-017-5199-5. arXiv:1706.00428 10.1140/epjc/s10052-017-5199-5 PMC6956957 31997920

[55] GEANT4 Collaboration, Geant4: a simulation toolkit. Nucl. Instrum. Methods A **506**, 250 (2003). 10.1016/S0168-9002(03)01368-8

[56] Nason P (2004). A new method for combining NLO QCD with shower Monte Carlo algorithms. JHEP.

[57] Frixione S, Nason P, Oleari C (2007). Matching NLO QCD computations with Parton Shower simulations: the POWHEG method. JHEP.

[58] Alioli S, Nason P, Oleari C, Re E (2010). A general framework for implementing NLO calculations in shower Monte Carlo programs: the POWHEG BOX. JHEP.

[59] Re E (2011). Single-top Wt-channel production matched with parton showers using the POWHEG method. Eur. Phys. J. C.

[60] Alioli S, Nason P, Oleari C, Re E (2009). NLO single-top production matched with shower in POWHEG: s- and t-channel contributions. JHEP.

[61] Frixione S, Nason P, Ridolfi G (2007). A positive-weight next-to-leading-order Monte Carlo for heavy flavour hadroproduction. JHEP.

[62] Czakon M, Mitov A (2014). Top++: a program for the calculation of the top-pair cross-section at hadron colliders. Comput. Phys. Commun..

[63] Campbell JM, Ellis RK (2010). MCFM for the Tevatron and the LHC. Nucl. Phys. B Proc. Suppl..

[64] CMS Collaboration, Performance of electron reconstruction and selection with the CMS detector in proton-proton collisions at $$\sqrt{s}=$$ 8 TeV. JINST **10**, P06005 (2015). 10.1088/1748-0221/10/06/p06005. arXiv:1502.02701

[65] D’Agostini G (1995). A multidimensional unfolding method based on Bayes’ theorem. Nucl. Instrum. Methods A.

[66] T. Adye, Unfolding algorithms and tests using RooUnfold. CERN-2011-006, 313 (2011). arXiv:1105.1160

[67] CMS Collaboration, Jet algorithms performance in 13 TeV data. CMS Physics Analysis Summary CMS-PAS-JME-16-003, CERN (2017). https://cdsweb.cern.ch/record/2256875

[68] CMS Collaboration, Precision luminosity measurement in proton–proton collisions at $$\sqrt{s} =$$ 13 TeV in 2015 and 2016 at CMS. Eur. Phys. J. C **81**, 800 (2021). 10.1140/epjc/s10052-021-09538-2. arXiv:2104.01927 10.1140/epjc/s10052-021-09538-2 PMC8550658 34781320

[69] CMS Collaboration, Measurement of the inelastic proton–proton cross section at $$ \sqrt{s}=13 $$ TeV. JHEP **07**, 161 (2018). 10.1007/JHEP07(2018)161. arXiv:1802.02613

[70] Lyons L, Gibaut D, Clifford P (1988). How to combine correlated estimates of a single physical quantity. Nucl. Instrum. Methods A.

[71] Valassi A (2003). Combining correlated measurements of several different physical quantities. Nucl. Instrum. Methods.

[72] Bansal M, Bansal S, Kumar R, Singh JB (2016). New observables for multiple-parton interactions measurements using Z+jets processes at the LHC. Phys. Rev. D.

[73] Y.-T. Chien et al., Precision boson-jet azimuthal decorrelation at hadron colliders (2022). arXiv:2205.05104

[74] HEPData record for this analysis (2022). 10.17182/hepdata.133278

